# An Exported Kinase Family Mediates Species-Specific Erythrocyte Remodelling and Virulence in Human Malaria

**DOI:** 10.1038/s41564-020-0702-4

**Published:** 2020-04-13

**Authors:** Heledd Davies, Hugo Belda, Malgorzata Broncel, Xingda Ye, Claudine Bisson, Viola Introini, Dominique Dorin-Semblat, Jean-Philippe Semblat, Marta Tibúrcio, Benoit Gamain, Myrsini Kaforou, Moritz Treeck

**Affiliations:** 1 Signalling in Apicomplexan Parasites Laboratory, The Francis Crick Institute, London, United Kingdom; 2 Division of Infectious Diseases, Department of Medicine, Imperial College London, London, UK,; 3 Institute of Structural and Molecular Biology, Birkbeck College, University of London, London, UK; 4 Cavendish Laboratory, University of Cambridge, Cambridge, United Kingdom; 5 Université de Paris, Biologie Intégrée du Globule Rouge, UMR_S1134, BIGR, INSERM, F-75015, Paris, France; 6 Institut National de la Transfusion Sanguine, F-75015, Paris, France; 7 Laboratory of Excellence GR-Ex, F-75015, Paris, France

## Abstract

The most severe form of human malaria is caused by *Plasmodium falciparum*. Its virulence is closely linked to the increase in rigidity of infected erythrocytes and their adhesion to endothelial receptors, obstructing blood flow to vital organs. Unlike other human-infecting *Plasmodium* species, *P. falciparum* exports a family of 18 ‘FIKK’ serine/threonine kinases into the host cell, suggesting that phosphorylation may modulate erythrocyte modifications. We reveal substantial species-specific phosphorylation of erythrocyte proteins by *P. falciparum*, but not by *Plasmodium knowlesi*, which does not export FIKK kinases. By conditionally deleting all FIKK kinases combined with large-scale quantitative phosphoproteomics we identify unique phosphorylation fingerprints for each kinase, including phosphosites on parasite virulence factors and host erythrocyte proteins. Despite their non-overlapping target sites, a network analysis reveals that some FIKKs may act in the same pathways. Only deletion of the non-exported kinase FIKK8 resulted in reduced parasite growth, suggesting the exported FIKKs may instead support functions important for survival within the host. We show that one kinase, FIKK4.1, mediates both rigidification of the erythrocyte cytoskeleton and trafficking of the adhesin and key virulence factor PfEMP1 to the host cell surface. This establishes the FIKK family as important drivers of parasite evolution and malaria pathology.

## Introduction

Humans are infected by at least five *Plasmodium* species that cause malaria, of which *Plasmodium falciparum* is responsible for the most debilitating form of the disease. Red blood cells (RBCs) infected by *P. falciparum* become more rigid and cytoadhere to the host vascular endothelium and other cells, avoiding clearance of infected cells by the spleen and leading to severe complications for the patient. Approximately 600 proteins are predicted to be exported into the host cell by *P. falciparum* to remodel the RBC ^2–4^. Cytoadhesion is mediated by a multigene family of *P. falciparum* Erythrocyte Membrane Protein 1 (PfEMP1), which is trafficked via membranous vacuoles in the RBC cytosol called Maurer’s clefts and inserted into protrusions on the RBC surface called knobs ^5^. Only one of the ~60 PfEMP1 variants is expressed at a given time, determining adhesion to a specific host receptor. For example, the gene *var2csa* encodes a PfEMP1 variant that binds to CSA on the placenta ^6^. Although other human-infecting Plasmodium species may cytoadhere^7^, they do not express PfEMP1, and the symptoms of infection are usually much milder in patients infected by these parasites.

**Figure 1:**
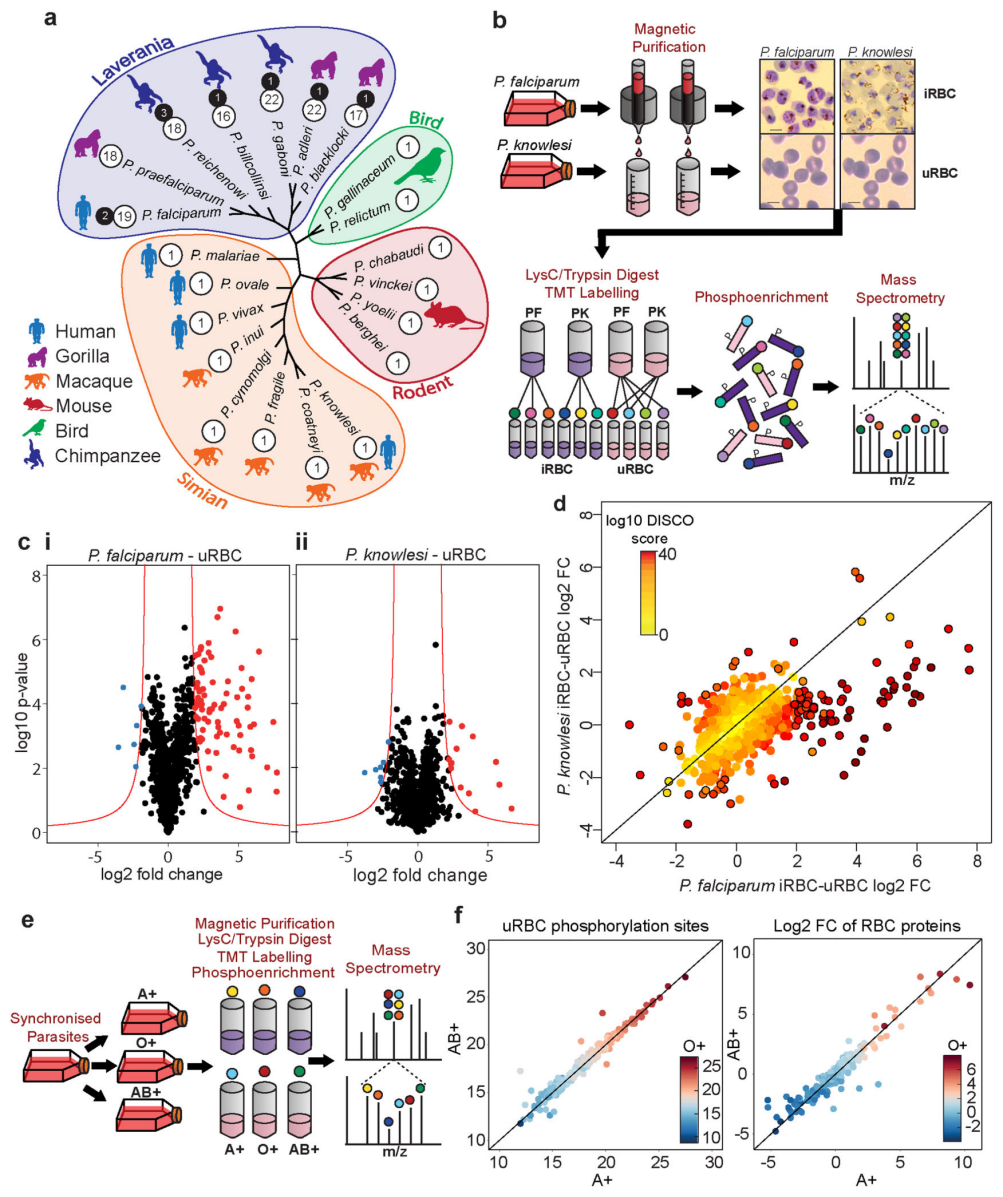
Infection of RBCs with *P. falciparum* or *P. knowlesi* induces species-specific changes to the phosphoproteome of RBC proteins independently of blood type. **(a)** Maximum likelihood phylogenetic tree of *Plasmodium* species, with clades grouped together. Silhouettes show host specificity. Divergence was calculated on the sequences of FIKK8 from each species. Numbers in white circles are the number of active FIKKs, black circles are pseudogenes. **(b)** Experimental workflow for phosphoproteomics of *P. falciparum* and *P. knowlesi*-iRBCs. Coloured circles represent the ten tandem mass tags. Scale bar - 5μm. **(c)** Volcano plots depicting the L2FC (x axis) in phosphosite intensity between uRBCs and RBCs infected with *P. falciparum* (i) or *P. knowlesi* (ii). A positive L2FC indicates that a protein residue is more phosphorylated in iRBCs than uRBCs. y axis = log10 *P*-value between technical replicates (Welch’s two-tailed *t*-test, n = 3). P – values were used to calculate technical variability but are not a direct indication of significance. Red line – non-linear significance threshold based on the log2 fold change and the -log10 p-value (see methods). **(d)** DISCO plot representing *P. falciparum*/uRBCs L2FC (y axis) vs *P. knowlesi*/uRBCs L2FC. Colour – discordance score (yellow - low, red – high, see methods). Circled points – phosphosites significantly changing upon infection with either *P. falciparum* or *P. knowlesi* **(e)** Experimental workflow for phosphoproteomics of *P. falciparum* in different blood types. **(f)** Comparison of the phosphoproteome of three different blood types, A+ (x axis), AB+ (y axis) and O+ (colour scale). Left – uRBC phosphorylation intensity, Right – L2FC in RBC protein phosphosite intensity between uRBCs and *P. falciparum*-iRBCs. See also Extended Data Figure 1 and Supplementary Dataset 3.

The FIKK kinases are a family of 18-26 serine/threonine kinases which are exported into the host cell by *Plasmodium* parasites of the *Laverania* clade, which includes *P. falciparum* and other great ape infecting species ^8–12^. Other *Plasmodium* species possess just one ancestral FIKK kinase, named FIKK8 in *P. falciparum*, which is not exported ^13^ (Figure 1A). The FIKKs contain a variable N-terminal region and a highly conserved kinase domain which lacks the canonical glycine-rich ATP-binding motif, but contains a Phe-Ile-Lys-Lys motif of unknown function located N-terminal to the kinase domain, for which they are named ^9^. Despite this atypical kinase domain, all FIKK kinases assayed so far have been demonstrated to be enzymatically active ^14–17^, and some have been implicated in modulating RBC properties ^16,18^, but their *in vivo* substrates remain unknown. Both RBC components and exported parasite proteins are phosphorylated during infection^19–28^; we hypothesise that the FIKK kinase family expanded to mediate these changes.

Here we compare human RBCs infected with two different malaria species, *P. falciparum* and *P. knowlesi*, and reveal substantial species-specific phosphorylation of RBC proteins almost exclusively in *P. falciparum* infected RBCs (iRBCs). Deep phosphoproteome profiling of knockout lines for all FIKK kinases predicted to be exported provides a detailed map of phosphorylation events controlled by each kinase, confirming that the FIKK kinases are key regulators of *P. falciparum*-specific changes to RBCs which are linked to severe malaria. Guided by the enrichment of phosphosites regulated by FIKK4.1 on cytoskeletal proteins and proteins involved in PfEMP1 trafficking, we demonstrate an important function for this kinase in controlling rigidity and cytoadhesion.

## Results

### Species-Specific Phosphorylation of RBC Proteins

The presence of exported FIKK kinases in *P. falciparum,* but not in other human-infecting *Plasmodium* species suggests that FIKK kinase activity plays a species-specific role in modulating RBC properties (Figure 1A). To test this, we compared the phosphoproteome of RBCs infected with *P. falciparum* to that of a *P. knowlesi* strain adapted for culture in human RBCs ^29^ (Figure 1B). As a control, we also determined the phosphoproteome of the uninfected RBCs (uRBCs) cultured under the same conditions. We purified late stage iRBCs at ~44 hours post infection (hpi) for *P. falciparum* and ~24hpi for *P. knowlesi*. Ten samples were analysed by mass spectrometry using Ten-plex Tandem Mass Tags (TMT) to quantify phosphosite intensity ^30^, including technical triplicates of *P. falciparum* and *P. knowlesi* iRBCs and uRBCs combined from both cultures, as well as one uRBC sample from the *P. falciparum* culture only.

We observed a substantial increase in ser/thr phosphorylation of RBC proteins in *P. falciparum* iRBCs compared to uRBCs (Figure 1C and Table S1). Using a threshold based on the log2 fold change (L2FC) and the *P*-value between replicates, 90 phosphosites showed a significant increase in phosphorylation upon infection by *P. falciparum* and 6 phosphosites showed a decrease in phosphorylation (Figure 1C panel i, and Table S3). Upon infection with *P. knowlesi*, only 20 phosphosites showed an increase in phosphorylation while 9 decreased (Figure 1C panel ii). To directly correlate the protein residues phosphorylated by the different species, we plotted the L2FC (iRBCs/uRBCs) for both *P. falciparum* and *P. knowlesi* in a concordance-discordance (DISCO) plot (Figure 1D). This illustrates that most residues are phosphorylated upon infection with *P. falciparum* only (Figure 1D, Table S3E). Notably, 53% of the residues phosphorylated by *P. falciparum* only have not been observed in uninfected RBC phosphorylation datasets previously (PhosphoSitePlus), indicating that these may be phosphorylated by *P. falciparum* FIKK kinases.

**Extended Data Fig. 1:**
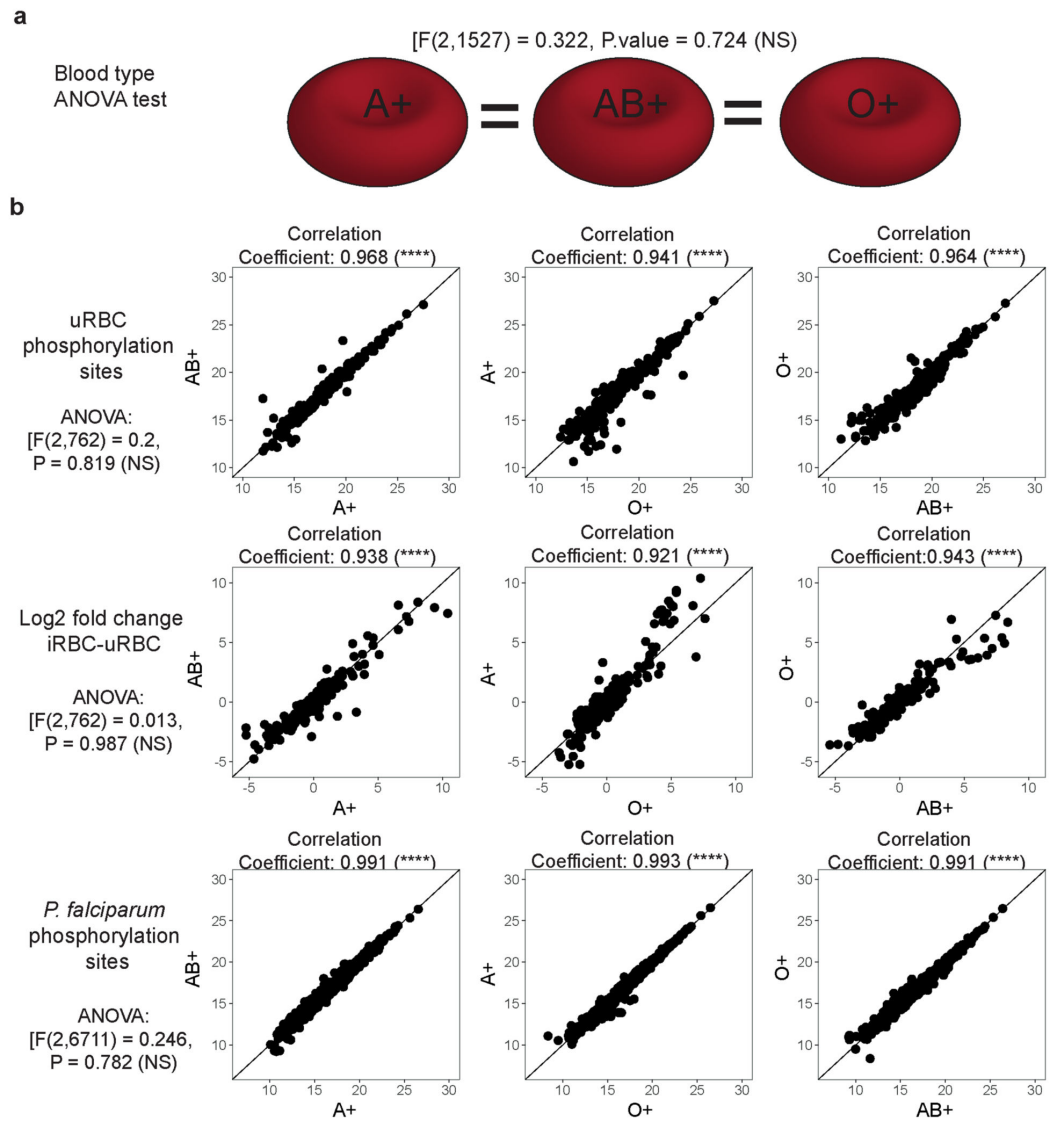
Statistical analysis of phosphorylation on RBC and *P. falciparum* proteins in different blood types. (a) One way ANOVA test. (blood type) Testing the hypothesis that blood type does not affect RBC phosphorylation in infected or uninfected samples (A+=AB+=O+). F-value: 0.322 ≫ 0.05, therefore blood type does not affect RBC phosphorylation. NS, not significant. N = 1 sample from each blood type. (b) Within-sample and species-specific testing of differences between blood types using one way ANOVA tests. Plots show phosphorylation intensity on uRBC proteins, log2 fold change on RBC proteins in iRBC-uRBC, and phosphorylation intensity on *P. falciparum* proteins, in two different blood types. There is no significant difference between blood types in any case (NS – not significant). Above each plot is the Pearson’s correlation coefficient (R) between the two blood types in the condition tested. There was no significant difference observed in any condition and there was a strong correlation between the phosphosite intensities in all blood types. **** - P ≤ 0.0001. N = 1 sample from each blood type.

To test whether phosphorylation of RBC proteins is affected by different host environments, we compared the phosphoproteome of *P. falciparum* cultured for one cycle in blood from three different donors with different blood types, A+, AB+ or O+ (Figure 1E). To ensure the parasites were collected at the same time points, we designed a magnetic purification stand with capacity for 5 medium-sized magnetic columns (MACS CS), based on a design by Kim *et. al* ^31^. We observed very few differences between the phosphoproteomes of the three blood types in either uninfected or infected RBCs (one way ANOVA testing [F(2,1527) = 0.322, *P*-value = 0.724 (NS)](Figure 1F, Extended Data Figure 1A, 1B and Table S3). This allows us to compare RBC phosphoproteomes across experiments where different blood samples are used.

### Systematic conditional deletion of FIKK kinases

**Extended Data Fig. 2:**
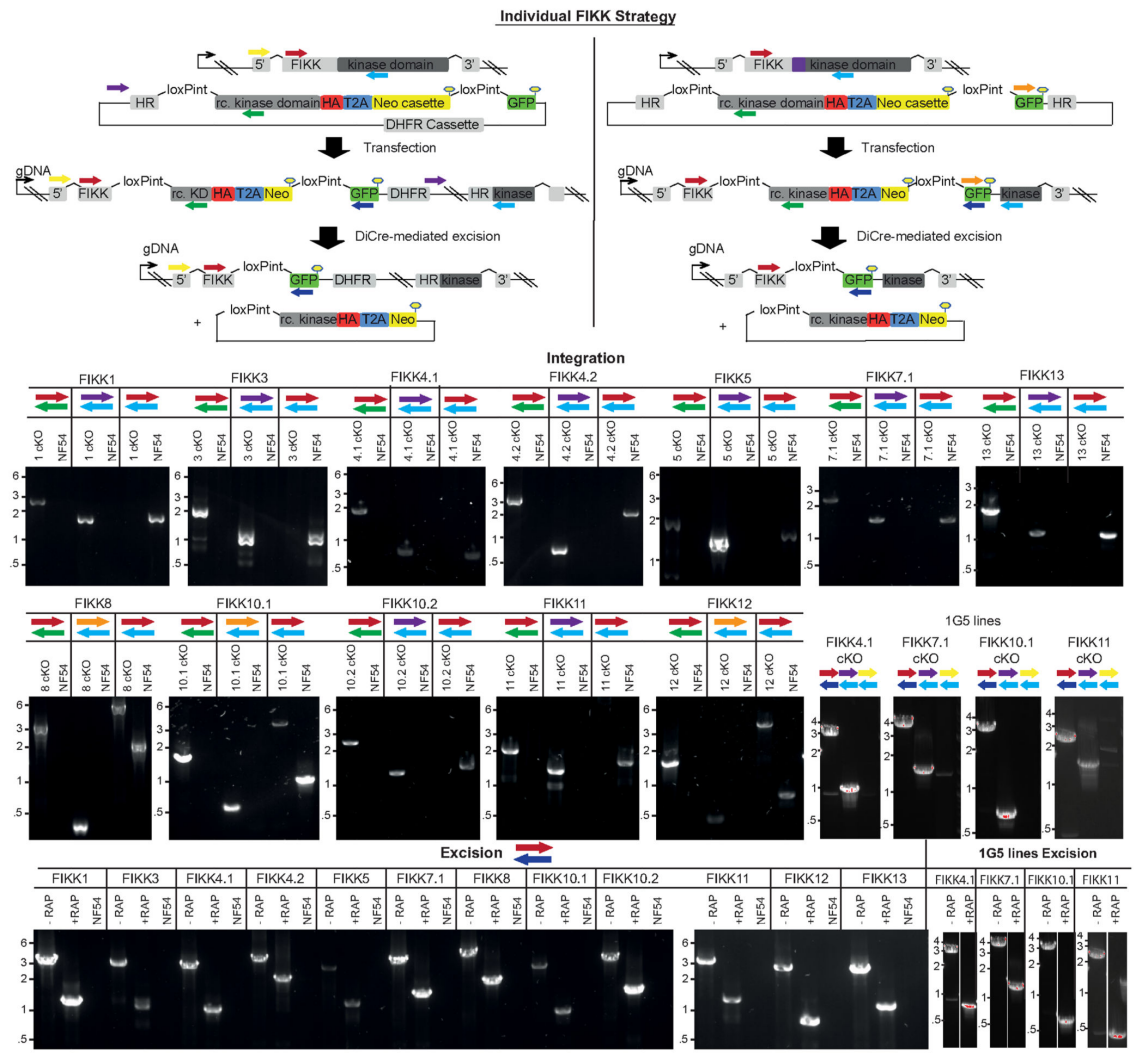
Correct integration of the different FIKK cKO plasmids into the respective endogenous loci and excision of their kinase domains upon RAP treatment. Schematics describing the different strategies used for generating the conditional FIKK knockout lines using selection linked integration or Cas9. Two LoxP introns were introduced on each side of the recodonised FIKK kinase domain, which was fused to a triple HA tag (red), a T2A skip peptide (blue) and a neomycin-resistance gene, to select for correct integration. Black arrows represent promoters and lollipops depict STOP codons. The relative positions of primers used to confirm correct integration of the plasmids into the respective loci and correct excision of the FIKK kinase domains upon RAP treatment are shown as coloured arrows. HR, homology region; Neo, neomycin-resistance cassette; RAP, rapamycin; rc. KD, recodonised kinase domain. Alongside the schematics are shown the PCR gels confirming correct integration and correct excision. DNA size markers in kbp are indicated on the left. PCRs for each FIKK were repeated at least 3 times with similar results for independent RAP-treatments.

**Figure 2:**
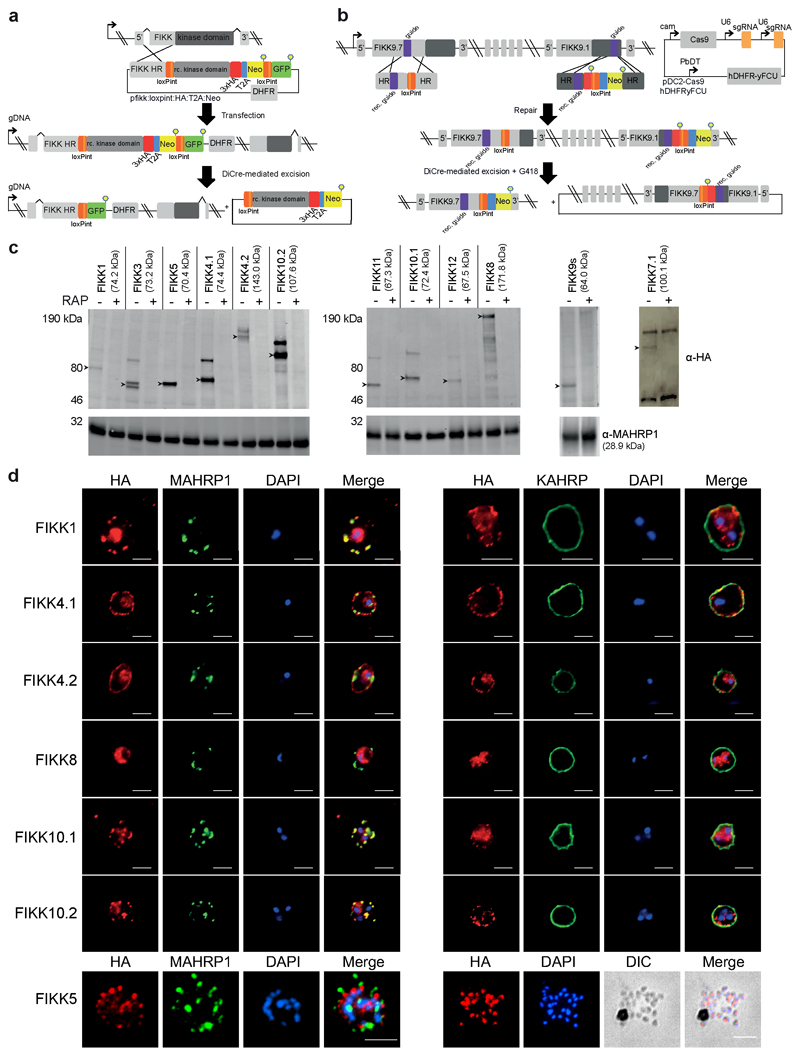
Conditional knockdown strategies for efficient excision and localisation of the FIKKs. **(a)** Description of the FIKK cKO strategy using the SLI method (Birnbaum, 2017). Schematic shows the structure of the *fikk* locus after integration and after Cre-mediated excision. **(b)** CRISPR/Cas9-mediated introduction of LoxPints into the *fikk9.1* and *fikk9.7* loci. RAP-treatment induces excision of all FIKKs located on chromosome 9 and expression of the Neomycin resistance cassette. **(c)** Western blots confirming correct expression and excision of FIKK kinases upon RAP-treatment. MAHRP1 antibody (bottom panel) demonstrates equal loading. FIKK7.1 samples were obtained by HA immunoprecipitation. Arrows – FIKK band at expected size (shown in labels). At least 3 independent western blots were performed for each FIKK with similar results. **(d)** Subcellular localisation pattern of a selection of FIKK kinases using antibodies against the C-terminal HA-tag fused to each FIKK kinase, MAHRP1 (Maurer’s cleft marker), and KAHRP (knob marker). DAPI was used for nuclear staining. For FIKK5, a late schizont is shown on the left panel, and a burst schizont on the right to demonstrate the localisation of the kinase in merozoites. Scale bar - 5μm. IFAs were performed at least 3 times for each FIKK with similar results.

We hypothesised that FIKK kinases may mediate the phosphorylation events specific to *P. falciparum* iRBCs. To identify the substrates of the FIKK kinases, we generated conditional gene knockouts (cKO) for all 19 FIKKs (excluding the pseudogenes FIKK7.2 and FIKK14). We targeted 12 FIKK kinases individually (FIKK1, 3, 4.1, 4.2, 5, 7.1, 8, 10.1, 10.2, 11, 12, 13) by flanking their kinase domains with two artificial introns containing LoxP sites (LoxPint) and introducing a C-terminal HA-tag in the same modification step, as previously described ^32^. Integration into the endogenous locus was either enhanced using a selection-linked integration (SLI) method ^33^ (Figure 2A), or by targeting the endogenous locus with CRISPR/Cas9 (Extended Data Figure 2).

**Extended Data Fig. 3:**
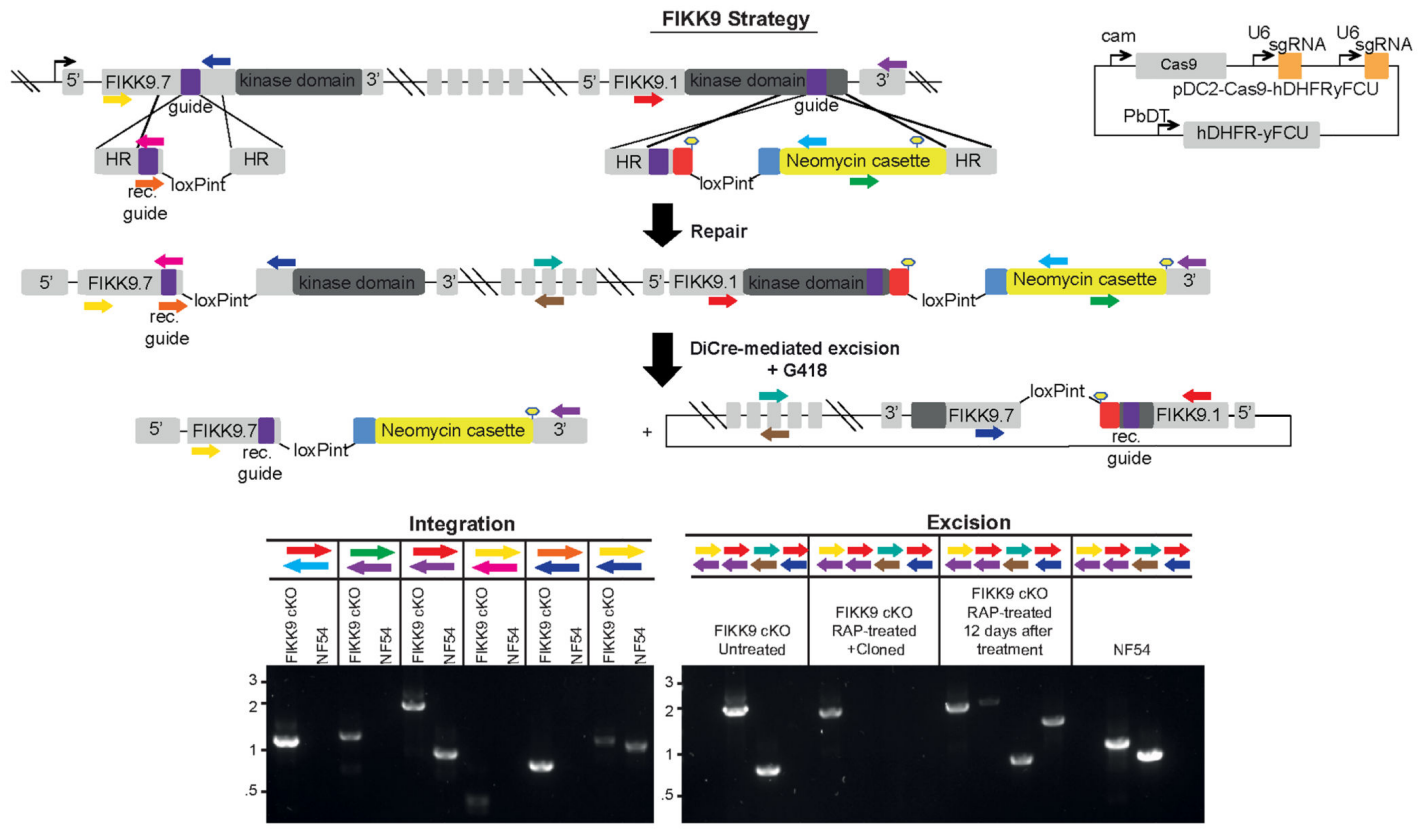
Integration of LoxP introns for the simultaneous excision of 7 FIKKs on chromosome 9 upon RAP treatment. For deletion of all 7 FIKKs on chromosome 9, the entire locus was flanked with LoxP introns. A triple HA tag (red) was inserted into FIKK9.1. A T2A skip peptide (blue) and neomycin resistance gene allow selection for correct excision. Black arrows represent promoters and lollipops depict STOP codons. The relative positions of primers used to confirm correct integration of the plasmids into the respective loci and correct excision of the FIKK kinase domains upon RAP treatment are shown as coloured arrows. HR, homology region; Neo, neomycin-resistance cassette; RAP, rapamycin; rc. KD, recodonised kinase domain. Alongside the schematics are shown the PCR gels confirming correct integration and correct excision. DNA size markers in kbp are indicated on the left. PCRs were repeated at least 3 times with similar results.

The remaining 7 FIKKs are located adjacently on chromosome 9. For these, we introduced two LoxPints into *fikk9.1* and *fikk9.7* genes in a single transfection step using a CRISPR/Cas9 strategy where two guide RNAs are expressed from a single plasmid (Figure 2B). To enhance the excision efficiency we introduced a Neomycin resistance cassette which would move into frame with the N-terminus of *fikk9.7* upon correct excision of *fikk9.1-9.7* (FIKK9s). All constructs were transfected into an NF54::DiCre line that allows for phenotyping in all lifecycle stages^34^. Five FIKKs (FIKK4.1, 7.1, 8, 10.1, 11) were additionally transfected into a 1G5::DiCre line ^35^. All parasite lines were cloned ^36^ and correct integration of constructs was verified by PCR (Extended Data Figure 2 and Extended Data Figure 3).

Western blot analysis of FIKK cKO lines showed HA-positive bands of various intensities at the predicted sizes (Figure 2C). FIKK7.1 was only detectable after concentration by HA-immunoprecipitation, and FIKK13 was not detectable under any conditions. We observed an additional protein band approximately 25kDa above the predicted size for most FIKKs, which is likely a result of incorrect skipping of the 2A peptide, leading to a minor population of a FIKK-Neomycin fusion protein. RAP-mediated excision of the FIKK kinases was confirmed by western blot and PCR 72h after treatment (Figure 2C and Extended Data Figure 2A). For the FIKK9 cluster, to ensure loss of the excised episome we selected for excised parasites using neomycin and obtained clones, for which we verify the absence of FIKK9s by western blot and PCR (Figure 2C and Extended Data Figure 3).

**Extended Data Fig. 4:**
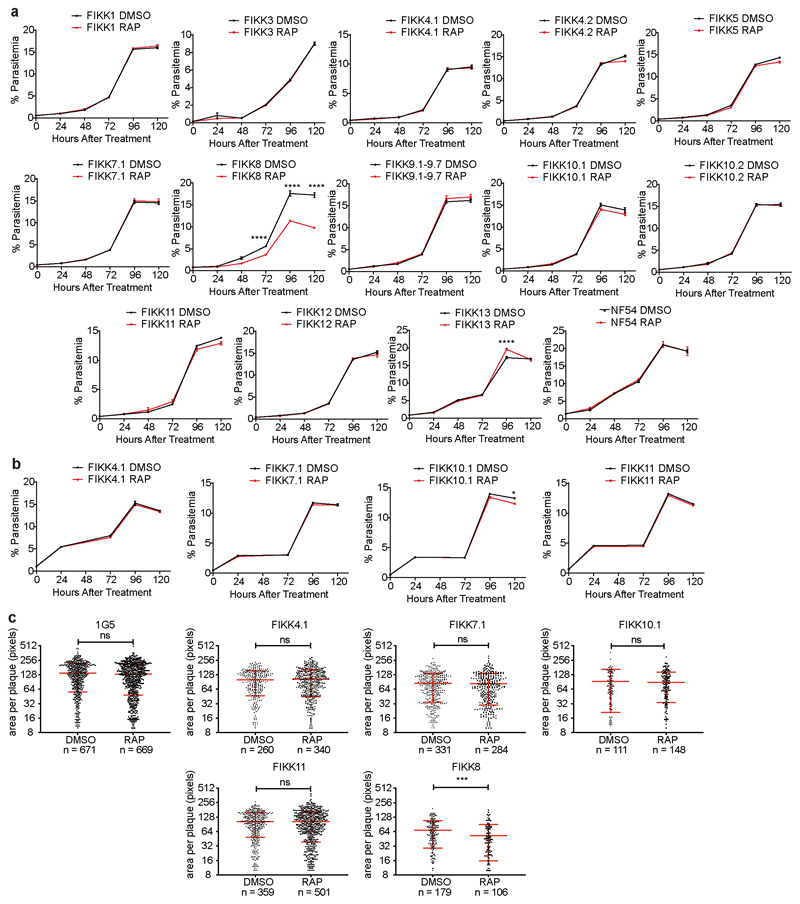
Exported FIKK kinases do not play a role in parasite growth under standard culture conditions. (a and b) Parasite growth curves for *Plasmodium falciparum* NF54 (a) and 1G5 (b) FIKK conditional knockout lines. Starting parasitemia was adjusted to 0.5% and samples were fixed every 24 hours for 120 hours (excluding at 48 h for the 1G5 lines). Parasitemia was measured by flow cytometry on 3 biological replicates for all FIKKs, and the mean and SEM are shown. Statistical analysis by two way ANOVA with Tukey correction for multiple comparisons. (**** p≤0.0001, * p ≤ 0.05, NS, not significant). Precise P values are shown in supplementary information table 2. (c) Scatter plots showing the area of plaques obtained by plaque assay for 1G5 FIKK conditional knockout lines, DMSO- (left) or RAP-treated (right). Horizontal bars indicate mean plaque area ± SD. Statistical significance was determined by a two-tailed *t*-test with no adjustment for multiple comparisons: 1G5 DMSO vs RAP (p=0.1626); FIKK4.1 DMSO vs RAP (p=0.4306); FIKK7.1 DMSO vs RAP (p=0.7201); FIKK10.1 DMSO vs RAP (p=0.5686); FIKK11 DMSO vs RAP (p=0.7912); FIKK8 DMSO vs RAP (p=0.0008) n = number of plaques, *** p<0.001, NS, not significant.

**Extended Data Fig. 5:**
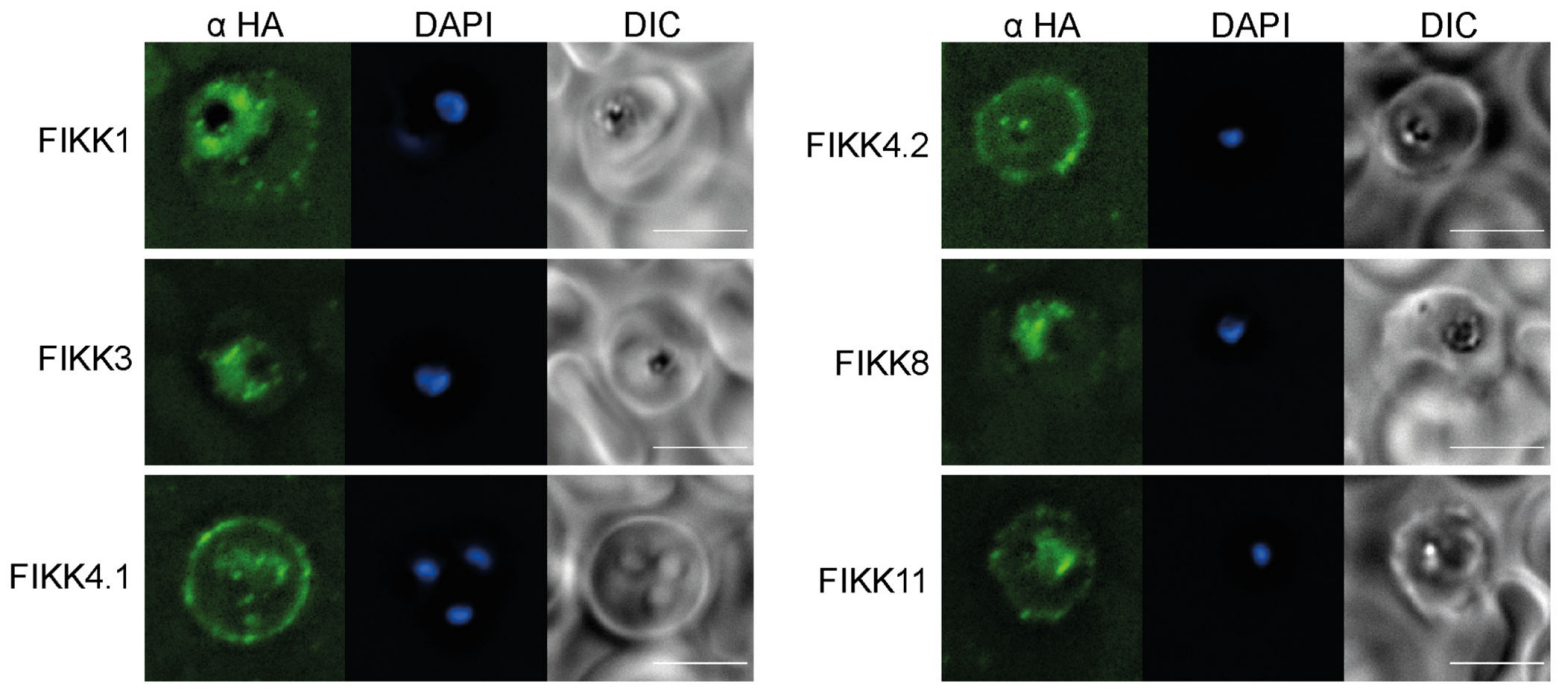
FIKKs are localised to different subcellular compartments Localisation pattern of additional FIKK kinases using antibodies against the C-terminal HA-tag fused to each FIKK kinase. DAPI was used for nuclear staining. The experiment was repeated at least 3 times independently for each FIKK with similar results. Scale bar - 5μm. See also Figure 2.

We next compared the growth rates of DMSO and RAP treated FIKK cKO lines along with the NF54::DiCre parental line by flow cytometry over 5 days. While for the non-exported FIKK8 we observed a significant drop in growth, no significant difference could be consistently observed for any exported FIKK lines over this time period (Extended Data Figure 4). The C-terminal HA-tag fused to each kinase allowed us to visualize their subcellular localisation by immunofluorescence assay (IFA). As expected, FIKK8 was not exported and localised within the parasite. Several FIKKs were clearly exported into the host cell, with some additional staining also observed within the parasite. Surprisingly, FIKK 3 and FIKK5 did not appear to be exported, with FIKK5 localising to merozoites. FIKK 1, FIKK10.1 and FIKK10.2 co-localised with the Maurer’s Cleft marker MAHRP1, whereas FIKK4.1, FIKK4.2 and FIKK11 localised to the RBC periphery, as predicted previously for FIKK4.1^37^ (Figure 2D and Extended Data Figure 5). FIKKs which showed very low expression levels by Western blot were also not detectable by IFA.

### FIKK kinases have unique phosphorylation fingerprints and are not redundant

**Figure 3:**
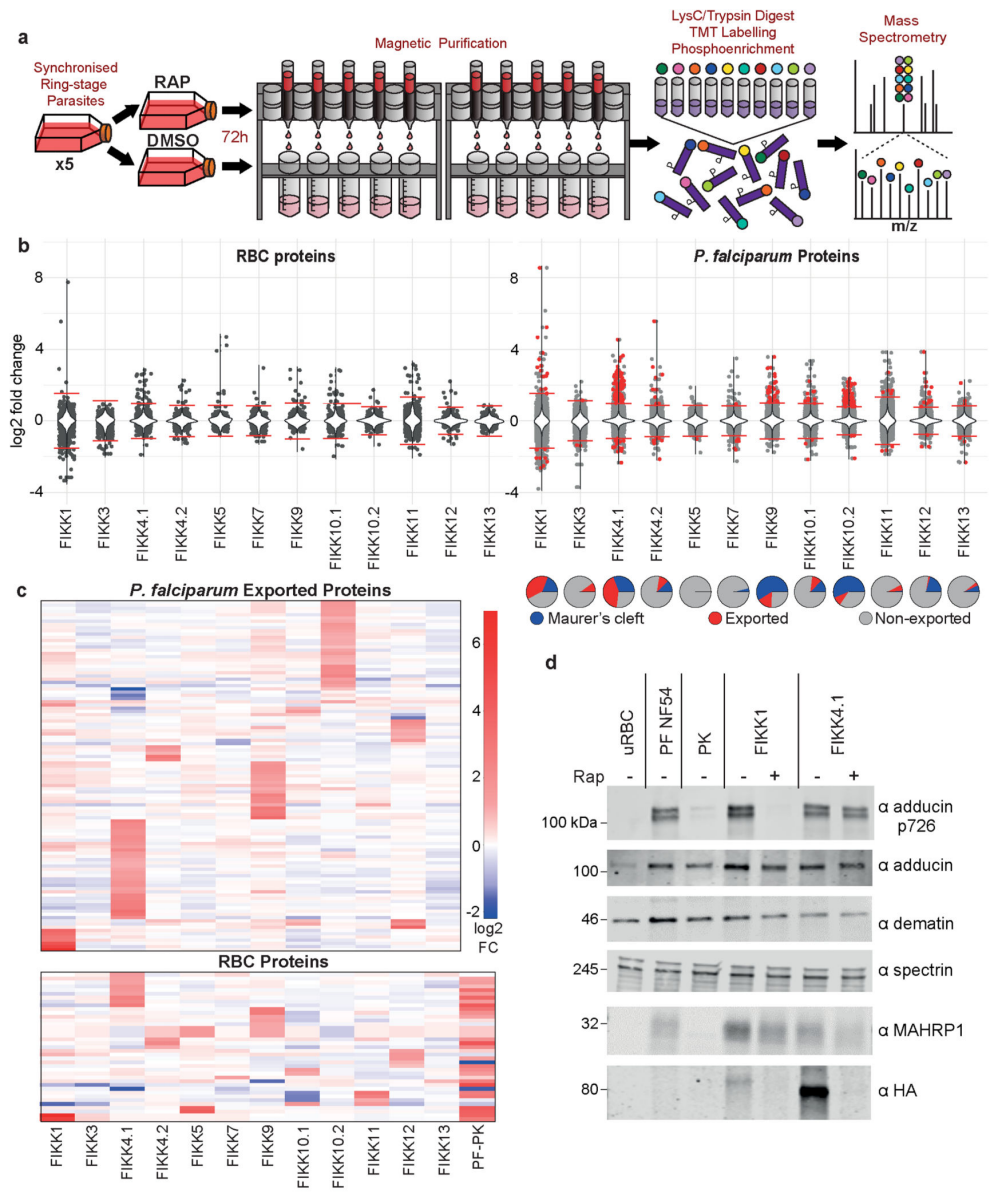
Each FIKK kinase influences the phosphorylation of distinct phosphosites on RBC and *P. falciparum* proteins. **(a)** Experimental workflow for phosphoproteomics of FIKK cKO lines. **(b)** Violin plots depicting the L2FC in phosphorylation intensity between DMSO and RAP-treated FIKK cKO lines, on RBC proteins (panel i) and *P. falciparum* proteins (panel ii). A positive L2FC indicates that phosphorylation is reduced upon FIKK-deletion. Red horizontal lines represent thresholds (4 x SD). Red points represent exported proteins which are significantly changing upon FIKK deletion. The violin plot represents the density and range of the data. n=2 for FIKK1, FIKK4.1, FIKK4.2, FIKK10.2 and FIKK11; n=1 for the other FIKKs. **(c)** Heatmaps representing the intensity of phosphosites identified in all four FIKK-deletion experiments which are significantly reduced upon deletion of at least one FIKK. *P. falciparum* exported proteins (top) and RBC proteins (bottom). The bottom panel includes the L2FC between *P. falciparum* and *P. knowlesi*-iRBCs across all experiments. Phosphosites (rows) are clustered by the complete linkage method with Euclidean distance measure. **(d)** Western blot confirming that adducin S726 is phosphorylated in *P. falciparum*-iRBCs and is FIKK1-dependent. Loading controls include α-adducin, dematin and spectrin for RBC loading, and MAHRP1 for *P. falciparum* infection. Anti-HA confirms excision of FIKK1 and FIKK4.1. Western blots were repeated at least 3 times using independent FIKK1 KO parasites with similar results. See also Supplementary Dataset 4.

To investigate the substrates of each FIKK kinase, we tightly synchronised the FIKK cKO lines and treated with either RAP or DMSO at ring stage. Late schizonts were enriched by magnet in the next cycle (Figure 3A). Four quantitative phosphoproteome experiments were performed (Table S1), two of which also included RBCs infected with the parental NF54 dicre line or *P. knowlesi*.

**Extended Data Fig. 6:**
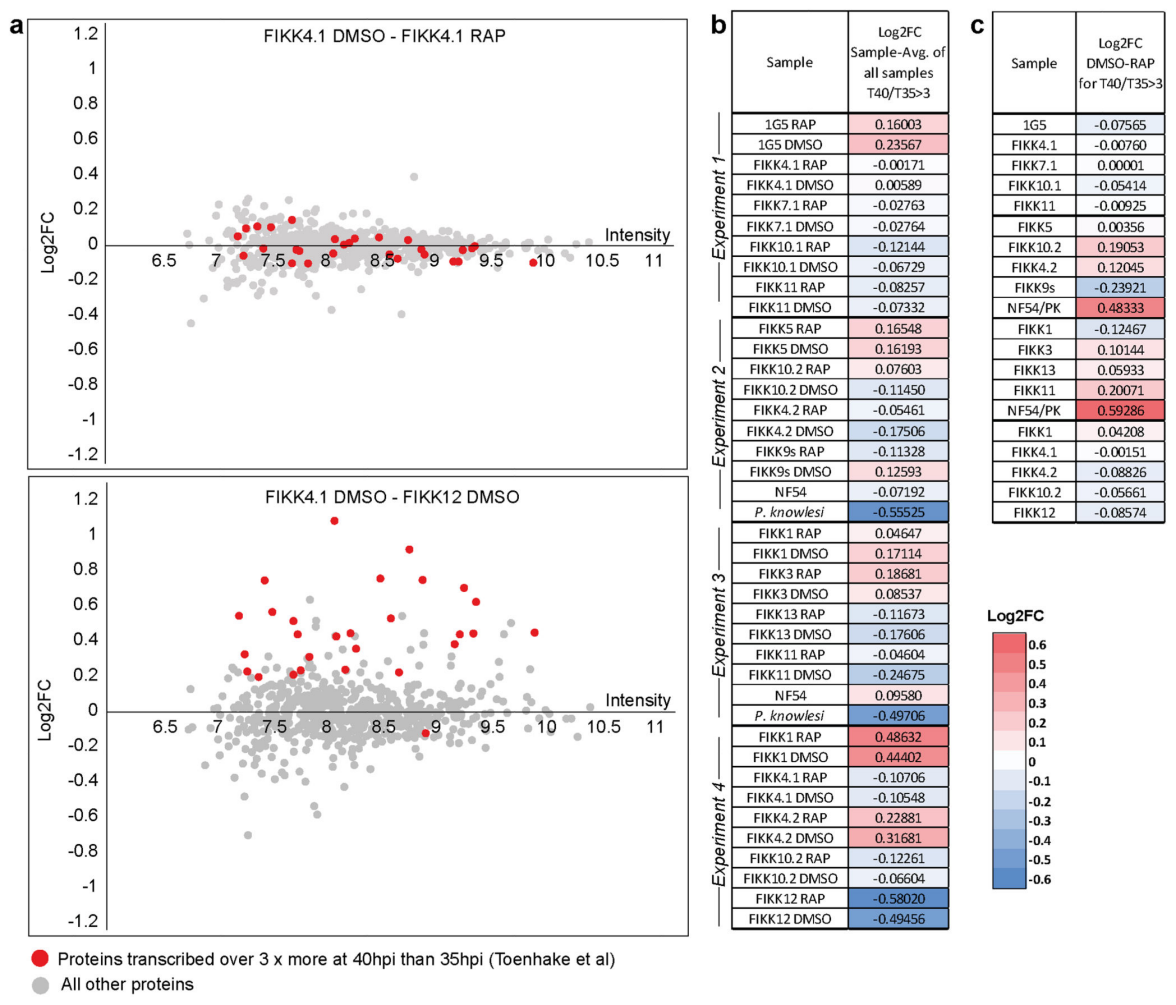
Unenriched proteome analysis of FIKK KO lines shows no difference in growth upon RAP-Treatment. (a) Plots showing the Log2 Fold change in protein intensity (y axis) between FIKK4.1 DMSO and FIKK4.1 Rap (Upper panel) and between FIKK4.1 DMSO and FIKK12 DMSO (lower panel), against the total intensity for all samples. Protein intensity was calculated from the average reporter intensity of all peptides on a protein, and only proteins for which more than 1 peptide was detected were included. In red are all proteins transcribed 3x more at 40 hpi than at 35hpi, according to RNA seq data 1. Between DMSO and RAP-treated lines the intensity of these proteins do not change substantially, while there is a clear increase in the FIKK4.1 DMSO line relative to FIKK12 DMSO, while all other proteins remain the same indicating a likely difference in growth. (b and c) To establish differences in growth between the lines, the log2 fold change in the intensity of the late-stage proteins between each line and the average of all lines within the experiment were calculated (b), or between RAP and DMSO-treated FIKK cKO lines (c). The log2 fold change for all other proteins was then subtracted from this to control for differences in protein abundance between samples.

Deletion of each FIKK kinase affected the level of phosphorylation on both RBC and *P. falciparum* proteins (Figure 3B and Table S4). To ensure that the observed differences were not due to changing protein levels we also analysed an unenriched proteome sample from each line (Table S2), and the L2FC in intensity of observed proteins between DMSO and RAP-treated samples are included in Table S3. While we did not observe all protein targets, almost no changes in phosphorylation could be explained by changes in protein levels (see supplemental discussion). By comparing the intensity of proteins expressed late in the parasite life cycle ^1^, we confirmed that while there was growth variation between the individual lines, the differences between DMSO and RAP-treated FIKK cKO lines were very small indicating that no growth delay occurred upon deletion of the FIKKs (Extended Data Figure 6). However, we did observe differences in growth between parasite lines, which is further discussed below.

The number of significantly changing phosphosites varied between FIKKs and generally correlated with the expression level of the kinase; deletion of highly expressed FIKK4.1 and FIKK10.2 resulted in the highest levels of differential phosphorylation while deletion of the barely detectable FIKK7.1 and FIKK13 affected very few phosphosites. For FIKK1, FIKK4.1, FIKK9s and FIKK10.2, more than half the residues less phosphorylated upon FIKK deletion are on proteins predicted to be exported into the host cell (Figure 3B, red circles). Furthermore, there is a clear pattern in the subcellular localisation of potential substrates, with deletion of FIKK10.2 and FIKK9s affecting mostly Maurer’s cleft proteins (Figure 3B, pie charts).

Unexpectedly, deletion of some FIKKs resulted in an increase in phosphorylation on several phosphosites, indicating indirect consequences of FIKK deletion. Deletion of FIKK4.1, FIKK4.2, FIKK9s, FIKK 10.1, FIKK10.2, and FIKK12 resulted in a reduction in phosphorylation on the kinase itself (FIKK9.3 for deletion of the FIKK9 cluster), either due to their truncation or loss of autophosphorylation. Interestingly, phosphorylation of FIKK10.2 was also dependent on FIKK1, suggesting one kinase may regulate the other, although the phosphorylated site is not within the kinase domain.

**Extended Data Fig. 7:**
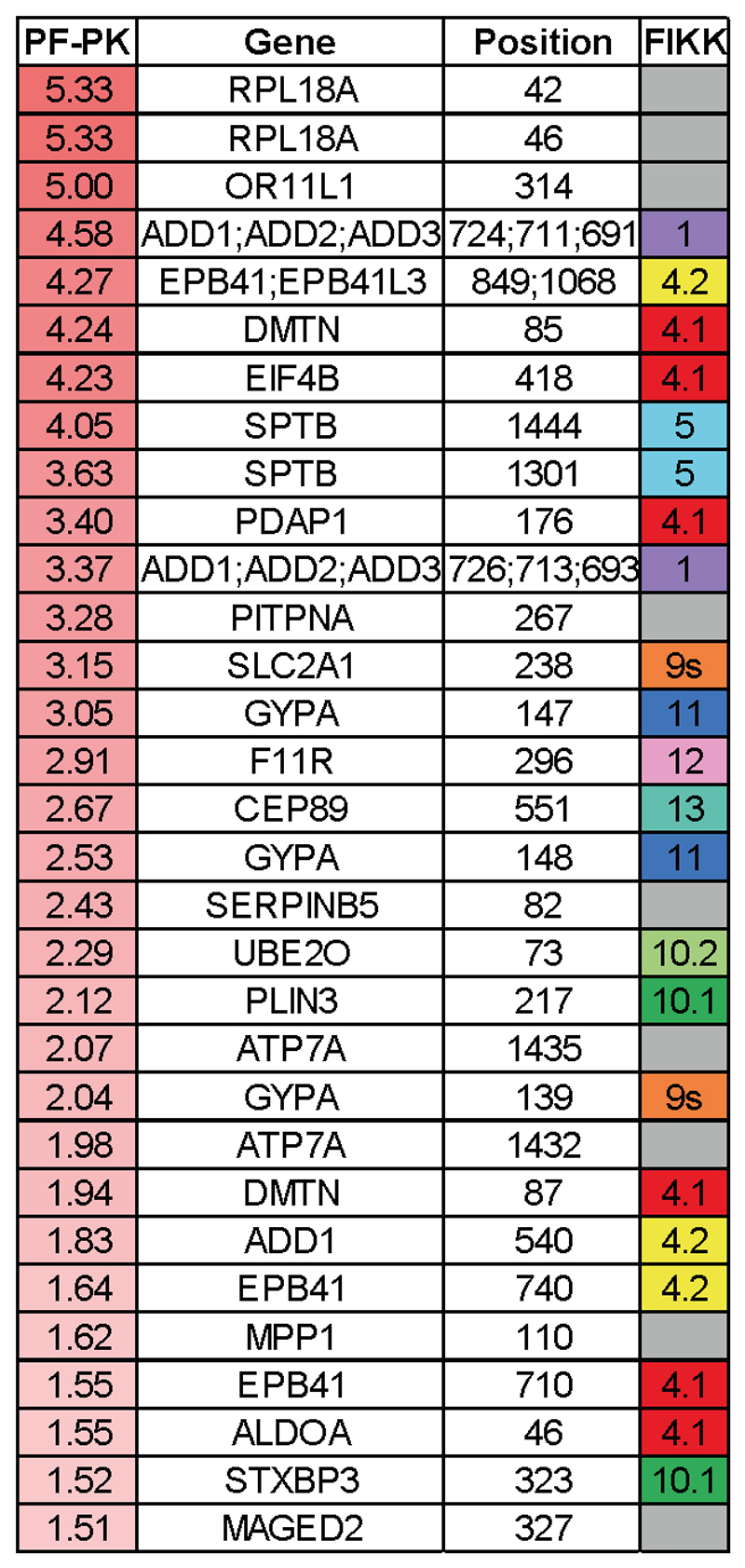
FIKK-dependent species-specific RBC phosphorylation. Phosphosites with the highest L2FC between *P. falciparum* and *P. knowlesi* (column 1) are labelled by any FIKK kinases which cause a significant reduction in phosphorylation of that site upon deletion (column 4).

To better visualise the relationships between the FIKK kinases, we assembled heatmaps representing the L2FC (DMSO/RAP) of phosphosites which were observed across all four datasets and which were significantly less phosphorylated upon deletion of at least one FIKK kinase (Figure 3C). A distinct fingerprint is observed for each FIKK kinase, demonstrating that FIKKs have largely non-overlapping functions. As expected, most FIKK-dependent phosphosites are not phosphorylated in *P. knowlesi* iRBCs. Of the 31 phosphosites significantly changing between RBCs infected by *P. falciparum* and *P. knowlesi* which were observed in all four experiments, 22 were also affected by FIKK deletion (Extended Data Figure 7), indicating that the export of FIKK kinases is primarily responsible for the difference in RBC phosphorylation between species.

Adducin S726 was previously observed to be phosphorylated upon infection by *P. falciparum*, but was believed to be a substrate of protein kinase C as it conforms to its preferred phosphorylation motif ^21^. Our data suggests that this is directly or indirectly mediated by FIKK1. We tested this by Western Blot using antibodies specific to adducin p726. The results, as predicted, showed near complete dependency of adducin S726 phosphorylation on FIKK1 (Figure 3D), supporting the validity of the mass-spectrometry data.

**Figure 4:**
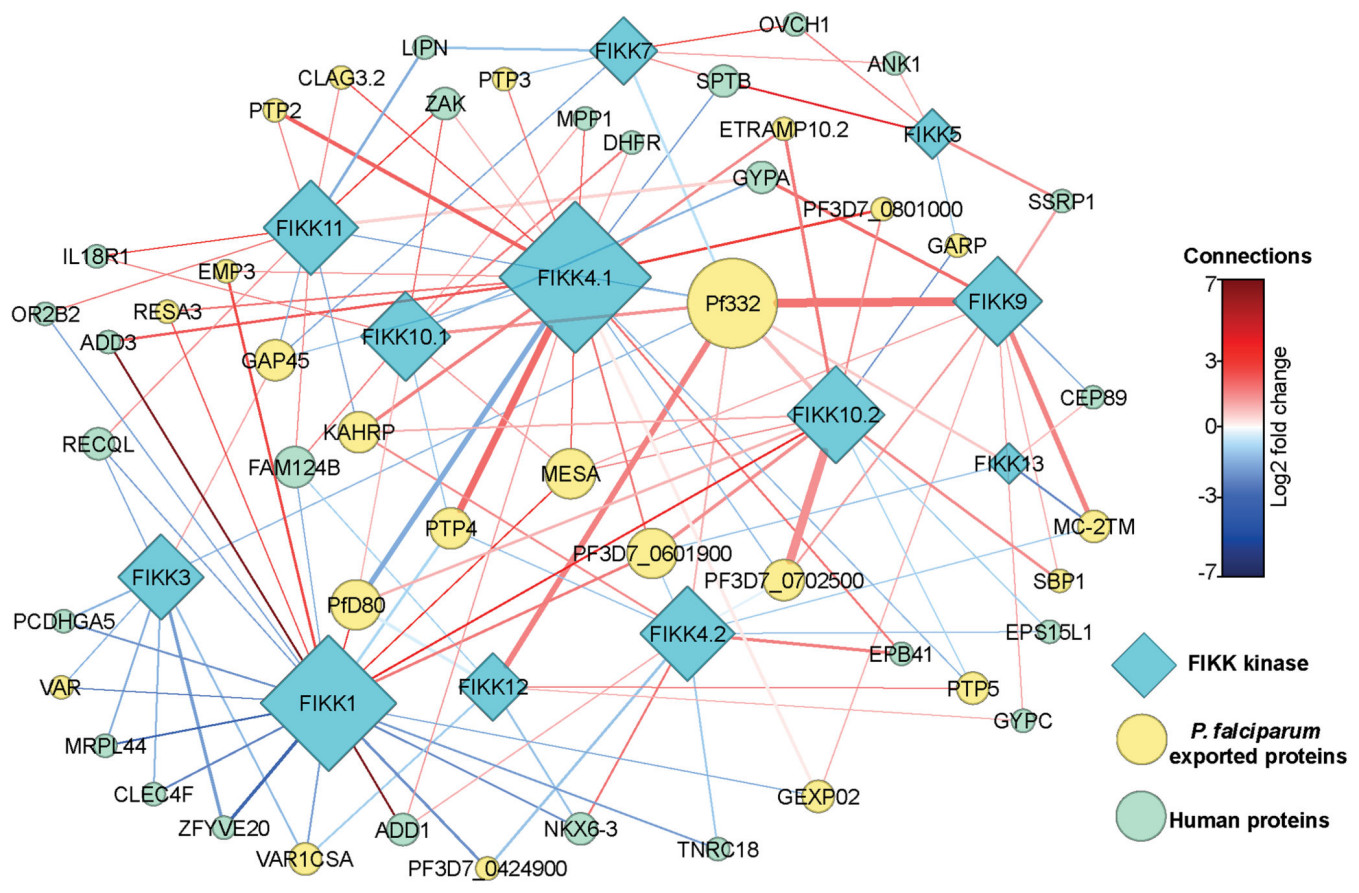
FIKK kinases are part of a complex network and act in synergy Network analysis of human RBC proteins (green) and *P. falciparum* exported proteins (yellow) which are significantly changing upon deletion of the FIKK kinases (blue). The thickness of the connecting lines represents the number of phosphosites significantly changing on each protein due to deletion of each kinase, while lines are coloured according to the average log2 fold change of all the significantly-changing phosphosites. Symbol size represents the number of connections to each protein.

We performed a network analysis to show the relationship between FIKKs acting on both human and exported parasite proteins (Figure 4). This revealed that some FIKKs are acting, directly or indirectly, on the same proteins. For example, deletion of 9 different FIKK kinases influences the phosphorylation of residues on Pf332, which is important for Maurer’s cleft morphology. Upon deletion of FIKK9s, 23% of the observed phosphosites on Pf332 are less phosphorylated, while the remaining phosphosites remain unchanged. Some phosphosites show opposite effects upon deletion of different kinases; for example, 5 out of 6 of the PfEMP1 trafficking proteins (PTP2, PTP3, PTP4, PTP5, and PTP6) are either more or less phosphorylated upon deletion of several FIKKs, indicating that PfEMP1 trafficking may be mediated by multiple kinases. Collectively, these data suggest that while FIKKs appear to target distinct phosphosites, they are part of a complex network and many FIKKs may act together in the same pathways.

### FIKK4.1 is important for modulating RBC rigidity and cytoadhesion

**Extended Data Fig. 8:**
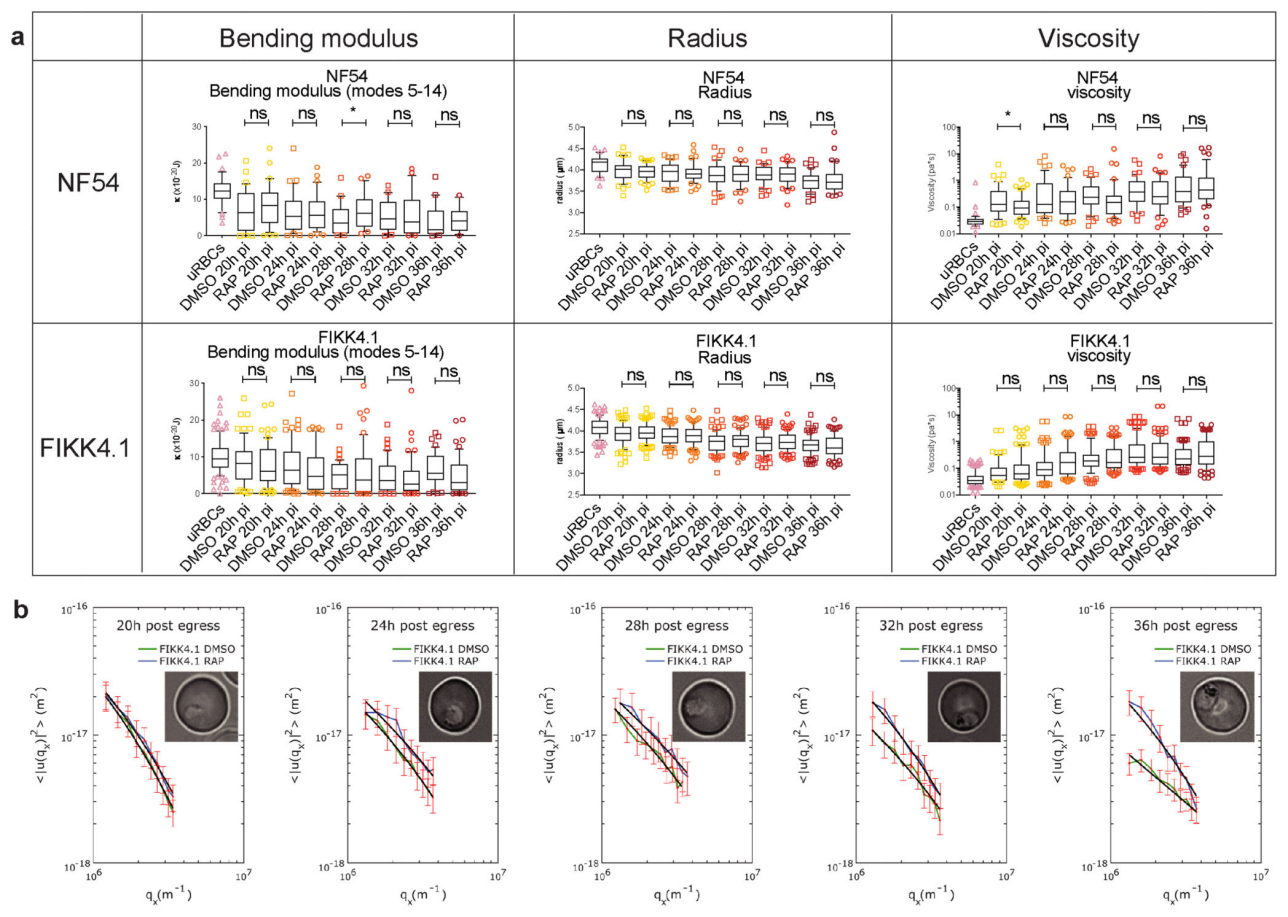
Flickering analysis of FIKK4.1 knockout. (a) Time-course flicker spectroscopy comparing the membrane bending modulus, the radius and the viscosity of uRBCs and RBCs infected with NF54::DiCre and FIKK4.1::DiCre DMSO- or RAP- treated parasites. Horizontal line within the box represents the median and whisker boundaries represent the 10^th^ and 90^th^ percentile. Points represent outliers. Statistical significance was determined by a two-tailed *t*-test. Precise p-values are included in supplementary information table 3. n = 2 biologically independent samples, the number of cells counted for each condition are summarised in supplementary information table 1. * p<0.05; NS, not significant. (b) Representative flickering spectra of DMSO- (in green) or RAP-treated (in blue) FIKK4.1::DiCre parasites at increasing time post-invasion. Mean square amplitude of fluctuations remains similar for DMSO-treated FIKK4.1::DiCre parasites throughout parasite development, while fluctuations in FIKK4.1 knockout parasites decrease significantly at 32 and 36 hours post-invasion. Fitted modes 5-14. The error bars are calculated as $SD/\sqrt{(n\times dt)/\tau_{q_{x}}},$ where *SD* is the standard deviation, *n* total number of frames (10000 frames per cell), *dt* time gap between each frame, and *τ_q_x__* the relaxation time for each mode. N = two biologically independent experiments.

**Figure 5:**
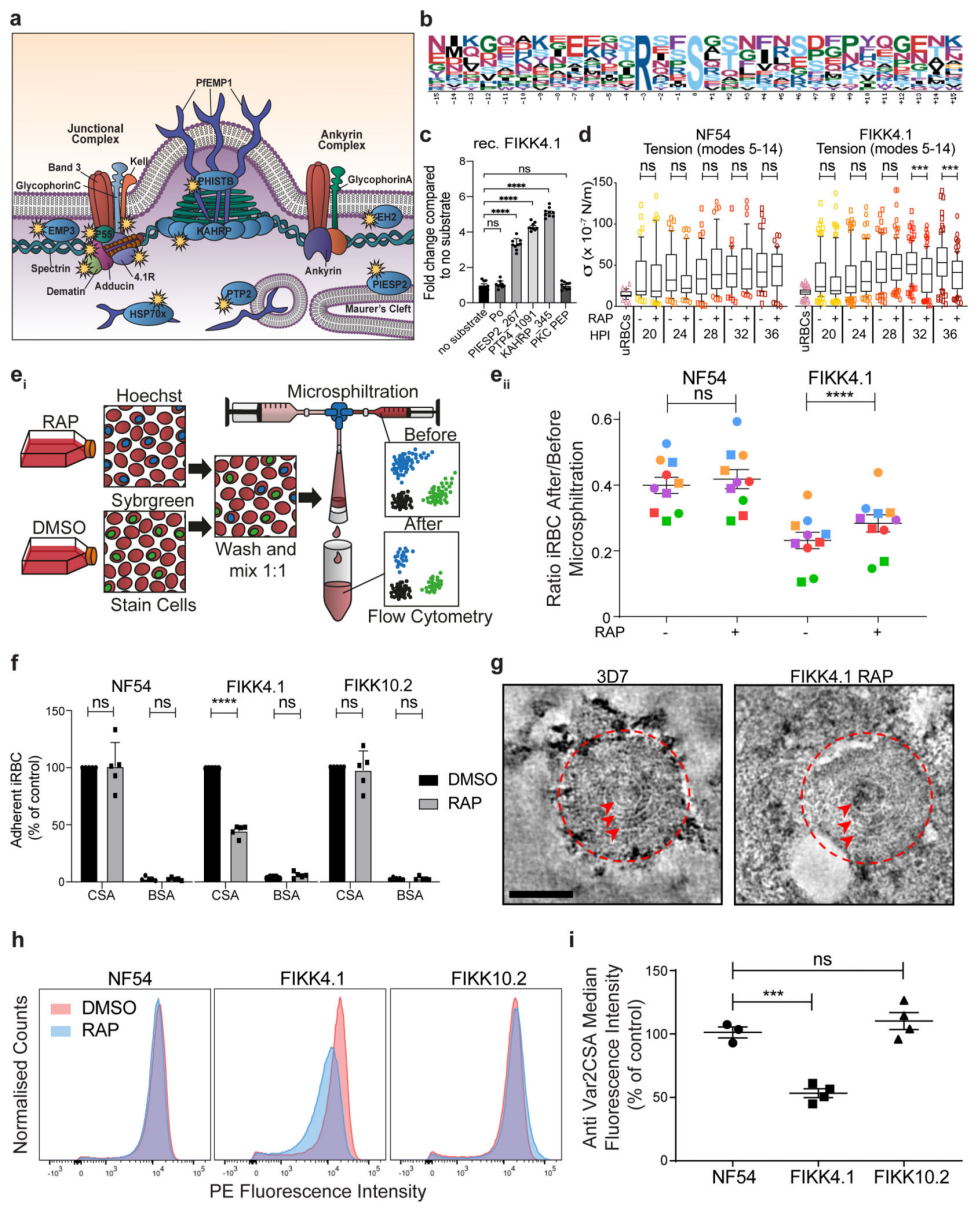
FIKK4.1 is important for the modulation of iRBC rigidity and PfEMP1 surface translocation. **(a)** Graphical representation of FIKK4.1 substrates identified by mass spectrometry (depicted with 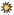). The representation focuses on the knob structures. **(b)** Arginine-based phosphorylation motif identified on FIKK4.1 substrates. See also Supplementary Dataset 5. **(c)** Recombinant FIKK4.1 activity on substrates Po, PIESP2_267, PTP4_1091, KAHRP_345 and PKC PEP. The results are represented in fold change compared to the no substrate luminescent signal obtained using the ADP-Glo assay ± SEM. Statistical significance was determined by one-way ANOVA followed by Tukey’s multiple comparison post-test: No substrate versus Po (p=0.9994); no substrate versus PIESP2_267 (p<0.0001); no substrate versus PTP4_1091 (p<0.0001); no substrate versus KAHRP_345 (p<0.0001); no substrate versus PKC PEP (p=0.9933). n = 3 biologically independent experiments each including 3 technical replicates. **** p<0.0001; NS, not significant. **(d)** Time-course flicker spectroscopy comparing the membrane tension of uRBCs, NF54::DiCre and FIKK4.1 cKO iRBCs, DMSO or RAP-treated. Horizontal lines within boxes represent the median and the whisker boundaries represent the 10^th^ and 90^th^ percentile. Points represent outliers. Statistical significance was determined by a two-tailed *t*-test: NF54 DMSO versus RAP 20hpi (p=0.8020); 24hpi (p=0.0884); 28hpi (p=0.1069); 32hpi (p=0.3869); 36hpi (p=0.5299). FIKK4.1 DMSO versus RAP 20hpi (p=0.3633); 24hpi (p=0.6115); 28hpi (p=0.9347); 32hpi (p=0.0001); 36hpi (p=0.0003). n = 2 biologically independent samples, the number of cells counted for each condition are summarised in Supplementary Information Table 1. *** p<0.001; NS, not significant. **(e_i_)** Diagram illustrating the microsphiltration experiment. **(e_ii_)** Ratio of iRBCs before and after microphiltration. Each point is an individual measurement with technical replicates from the same experiment represented in the same colours. Error bars show mean ± SEM, n = 10. FIKK4.1 DMSO vs RAP (**** p<0.0001), NF54 DMSO vs RAP (NS, not significant; p=0.0811) by paired multiple comparison one-way ANOVA with Sidak correction. **(f)** Percentage of iRBCs bound to CSA or BSA compared to the positive control (DMSO-treated parasites on CSA). Shown is the mean cytoadhesion compared to control ± SEM. The results were statistically tested with a two-way ANOVA test plus a multiple comparison Sidak test comparing all means: NF54 CSA DMSO versus RAP (p=0.9802); NF54 BSA DMSO versus RAP (p=0.9997); FIKK4.1 CSA DMSO versus RAP (p<0.0001); FIKK4.1 BSA DMSO versus RAP (p=0.5021); FIKK10.2 CSA DMSO versus RAP (p=0.8704); FIKK10.2 BSA DMSO versus RAP (p>0.9999) n = 5 biologically independent samples, each with at least two technical replicates, which were averaged before statistical analysis. **** p<0.0001; NS, not significant. **(g)** Typical knob complexes from wild-type 3D7 and FIKK4.1 KO schizonts imaged by negative stain electron tomography. The knob coat is outlined with a red dashed line and the underlying knob spiral is indicated by red arrowheads. Side views of the knobs are also available in Extended Data Figure 9C. Images are an average of 5 central slices of the tomogram (scale bar - 50 nm). The experiment was performed once with a representative cell selected from at least 10 observed cells. **(h)** Normalised flow cytometry histograms showing PE fluorescence, which reflects the amount of Var2CSA on the cell surface for each line. The experiment was repeated at least 3 times with similar results. **(i)** Quantification of the VAR2CSA median fluorescence intensity in RAP-treated parasites compared to the control (DMSO-treated) ± SEM, n = 3 for NF54, n = 4 for FIKK4.1 and FIKK10.2. NF54 vs FIKK4.1 (***; p=0.0005, NF54 vs FIKK10.2 (NS, not significant; p = 0.4691) by multiple comparison ANOVA).

A substantial concentration of the potential substrates of FIKK4.1 are on proteins known to be important for modulating cytoadherence to host receptors and the formation of knobs^38–45^. Additionally, phosphorylation of several proteins from the 4.1R complex of the erythrocyte cytoskeleton is dependent on FIKK4.1 (Figure 5A and Table 1). We identified a very strong enrichment for a basic phosphorylation motif with Arginine in the -3 position for FIKK4.1-dependent phosphosites (Figure 5B). A basic phosphorylation motif has been also identified for FIKK8 using an artificial substrate called *Po* (RRRAP**S**FYRK) ^15^. To test whether FIKK4.1 can phosphorylate some of the identified substrates directly, we performed kinase assays using synthetic peptides from PIESP2 (PIESP2_267: EIRQE**S**RTLIL), PTP4 (PTP4_1091: HTRSM**S**VANTK) and KAHRP (KAHRP_345: GSRYS**S**FSSVN), which all contain an Arginine in a -3 position relative to a Serine. All predicted substrates are phosphorylated by FIKK4.1 (Fig. 5C) however it was unable to phosphorylate the peptide Po (RRRAP**S**FYRK)^15^ or the protein kinase C synthetic peptide substrate PEP (QKRP**S**QRSKYL)^46^, which contains a basic lysine in the -3 position (Fig. 5C). This suggests that the Arginine residue in the -3 position may be necessary but is not sufficient for phosphorylation by FIKK4.1, and that the FIKKs differ in their substrate preferences. The 4.1R protein complex tethers spectrin tetramers together and links the plasma membrane to the underlying cytoskeleton ^47^. To test whether phosphorylation of this complex by FIKK4.1 affects the structural properties of the RBC, we first performed membrane fluctuation analysis (flicker spectroscopy) ^48,49^ of RBCs infected with NF54::DiCre or FIKK4.1 cKO parasites treated with either DMSO or RAP. As described previously, a gradually increasing rigidification of the iRBC membrane is observed ^49^, however the FIKK4.1-deleted parasites were less rigid than the DMSO control from 32 hours post invasion onwards (Figure 5D, mean reduction in membrane tension = 25.10% at 32hpi, 26.94% at 36hpi, see Table S7 for the number of cells counted for each condition) demonstrating that indeed FIKK4.1 is important for modulating RBC rigidity. None of the other parameters measured by flickering analysis (bending modulus, radius or viscosity) changed upon FIKK4.1 deletion (Extended Data Figure 8A and B) confirming that FIKK4.1 acts specifically on the RBC cytoskeleton and not on the composition of the RBC membrane, and that there is no growth difference between the lines ^49^.

Rigidification of RBCs leads to increased retention in the spleen which can be mimicked *in vitro* by microsphiltration assays ^50^. To further support the phenotype observed by flickering analysis at a time point where differences were observed, we performed microsphiltration experiments on the parasites between 30 and 34hpi. We labelled RAP-or DMSO-treated parasites with either SYBR green or Hoechst, before combining them in a 1:1 ratio and measuring the parasitemia before and after microsphiltration by flow cytometry (Figure 5E panel i). RAP-treated FIKK4.1 showed a 19.5% increase in the number of parasites passing through the beads (Figure 5E panel ii. Mean increase in the ratio of iRBCs after/before microsphiltration between DMSO and RAP = 19.5%±2.4 %; mean±SEM; n=10, including 2 technical replicates in 5 experiments), indicating a reduction in rigidity. No differences were observed for the NF54 parental line, although it was consistently less rigid than the FIKK4.1 line overall (see supplementary discussion). Collectively these data provide strong support for an important but non-exclusive role of FIKK4.1 in modulating RBC rigidity.

**Extended Data Fig. 9:**
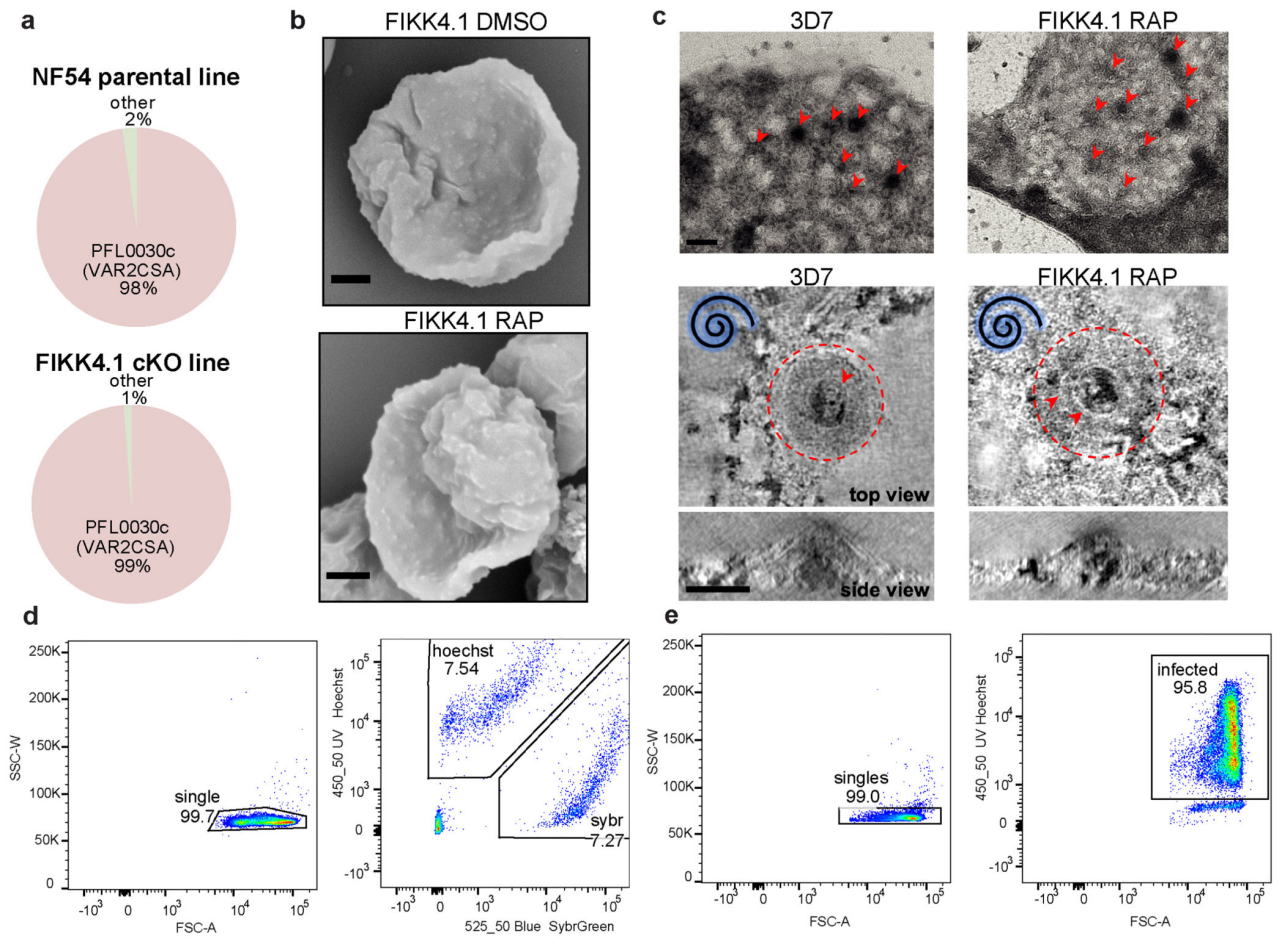
Cytoadhesion and Var2CSA surface translocation of FIKK4.1 knockout. (a) *Var* gene transcription profile of NF54::DiCre and FIKK4.1::DiCre parasites determined by qPCR. Transcriptional levels of each *var* genes were normalized with the housekeeping gene, seryl-tRNAtransferase. (b) SEM images of the surface of erythrocytes infected with DMSO or RAP-treated FIKK4.1::DiCre parasites. The experiment was repeated 2 times with similar results (scale bar - 1μm). (c) Top panels: Electron micrographs of knobs on detergent treated, negatively-stained RBC ghosts from wild-type 3D7 and FIKK4.1KO schizonts imaged at low-magnification. Red arrows indicate the position of knobs, which show up as circular _dark_ patches on the membrane (scale bar - 200 nm). Bottom panels: Negative-stain electron tomography reveals typical structural features of the knob complex. In top views of knobs (XY), the knob-coat is outlined with a red dashed line and the underlying knob spiral is indicated by red arrow heads. In these examples of knobs, the underlying spiral is left-handed (indicated by the blue spiral symbol) showing that the knob is pointing upwards from the plane of the grid. A side view (XZ) of the same knob shows that the height and diameter of the knobs in wild-type 3D7 schizonts FIKK 4.1KO schizonts is similar. Knobs with a right-handed spiral are shown in Figure 5g. These knobs are pointing downwards and are compressed against the surface of the grid, hence more rings of the spiral structure are visible in the plane of the tomogram. Images are an average of 5 central slices of the tomogram (scale bar - 50 nm). (d) Gating strategy for microsphiltration flow cytometry experiments. (e) Gating strategy for Var2CSA surface expression experiments – the histograms shown in figure 5h include all infected cells, and the median PE- fluorescence is calculated on this population. A similar gating strategy was used for growth curves using Hoescht or Sybrgreen.

The enrichment of proteins important for PfEMP1 surface translocation and knob formation among the predicted substrates of FIKK4.1 indicated that these processes may also be affected by FIKK4.1 deletion. qPCR shows that almost all parasites within the population expressed *var2csa,* the PFEMP1 variant which binds CSA ^51^(Extended Data Figure 9A). This allowed us to test cytoadhesion in petri dishes coated with CSA or BSA as a control. Upon deletion of FIKK4.1, a 55% reduction in cytoadhesion was observed relative to the DMSO control (mean reduction in cytoadhesion = 55.01±1.94%; mean±SEM; n=5) (Figure 5F). No significant differences were observed for RAP-treated NF54 or FIKK10.2.

As the major knob component KAHRP is differentially phosphorylated upon FIKK4.1 deletion, the reduction in cytoadhesion may result from a defect in knob formation. However, scanning electron microscopy (SEM) did not reveal any obvious abnormality in knobs upon FIKK4.1 deletion (Extended Data Figure 9B). Likewise, negative stain electron tomography ^52^ on RBC ghosts did not reveal any differences in the size, shape, distribution, or structural features of knobs between FIKK4.1 cKO iRBCs compared to 3D7 wildtype parasites (Figure 5G and Extended Data Figure 9C), suggesting that FIKK4.1 does not play a role in knob architecture. This indicated that PfEMP1 trafficking to the surface may be affected. Quantification of surface exposed VAR2CSA by flow cytometry revealed a 46.7% reduction in the median fluorescence intensity observed in RAP-treated FIKK4.1, but not in NF54::DiCre or FIKK10.2 parasite lines (mean reduction = 46.7±3.04%; mean±SEM; n=4) (Figure 5H and I). As PfEMP1 was still observed to some extent on the surface of most iRBCs, it is likely that a reduction in avidity due to fewer PfEMP1-CSA interactions is responsible for the cytoadhesion defect upon FIKK4.1 deletion.

## Discussion

Phosphorylation of *P. falciparum*-infected RBC has previously been reported ^20–22,26^, however, the kinases that mediate the phosphorylation events remain largely unexplored. Here, using extensive phosphoproteomic profiling of FIKK kinase KOs and RBCs infected with two different *Plasmodium* species we greatly expand the infection -induced phosphoproteome of the RBC and show that FIKK kinases are species-specific effector proteins important for a substantial fraction of infection-induced phosphorylation in the RBC. The unique phosphorylation fingerprint of each FIKK kinase demonstrates that while some FIKKs may operate in the same pathways, each has an independent role.

The expansion and subsequent conservation of the FIKK family in the *Laverania* strongly argues for an important role in modulating host-pathogen interactions ^8,11^. The vital role of FIKK4.1 in PfEMP1-mediated adhesion to CSA supports this hypothesis, but it is likely that PfEMP1 trafficking is not the only evolutionary driver of FIKK expansion. Other Laverania-specific traits include an extended sexual development process^53–55^, where FIKKs may play a crucial role. As all cKO lines were made in an NF54::Dicre line capable of transmitting through mosquitoes ^34^, it will be possible to test their roles across all life stages in the future. Species-specific RBC phosphosites are observed on proteins predicted to play a role in cytoskeletal connections, nutrient permeability, and the ubiquitination system, all of which have been previously reported to be modulated during *P. falciparum* infection^23,25,56–66^ and our analysis indicates that FIKKs may regulate these pathways, either directly or by co-opting host kinases^24,67,68^. Although no consistent growth defect was observed upon deletion of any exported FIKK kinase after 120 hours under optimal cell culture conditions, a recent genetic screen indicated that disruption of some FIKKs may have a fitness defect over a longer time scale ^69^, and some FIKKs were refractory to non-conditional gene deletion ^70^. Additionally, FIKK9.3 has been implicated in providing some protection against elevated temperatures, suggesting it may be important during fever ^71^.

As we demonstrate for FIKK4.1, knowing the substrates of these FIKKs can provide a mechanistic understanding of species-specific disease outcomes. Although human infections with FIKK deletion lines will be required to ultimately prove their role in regulating virulence traits, our results imply an important role for FIKKs in the pathogenesis of human malaria. By blocking the activity of multiple FIKK kinases simultaneously using kinase inhibitors, it may be possible to prevent RBC remodelling entirely to ease disease symptoms and enable rapid clearance of infected cells by the host immune system.

## Material and Methods

### Phylogeny

The protein sequences of FIKK8 orthologues from all available *Plasmodium* species were downloaded from PlasmoDB (2018) ^72^. Multiple sequence alignment was performed with ClustalW ^73^, then maximum likelihood-based phylogeny was calculated by PhyML 3.0 ^74^. The phylogenic tree was visualized with FigTree v1.4.4 ^75^ using a radial tree layout with branches transformed to equal length for clarity.

### *In vitro* maintenance and synchronization of parasites

Asexual RBC stage *P. falciparum* parasites were cultured at 37°C in complete media (CM). Complete media consists of RPMI-1640 medium which was supplemented with 5g Albumax II (ThermoFischer Scientific) to act as a serum substitute, 0.292g L-glutamine, 0.05g hypoxanthine, 2.3g sodium bicarbonate, 0.025g gentamicin, 5.957g HEPES and 4g dextrose. Parasites were grown at 1-5% haematocrit; blood was from anonymous donors, provided through the UK Blood and Transfusion service. Parasites were grown in a parasite gas atmosphere (90% N_2_, 5% CO_2_, 5%O_2_) ^76^. Parasite growth was routinely examined by Giemsa staining of methanol-fixed air-dried thin blood smears followed by visualisation by light microscopy. Asexual RBC stage *P. knowlesi* parasites were cultured in CM supplemented with 10% human serum as described previously ^29^. Parasite cultures were synchronised by isolating mature schizont-stage parasites on a cushion of 60% Percoll (GE Healthcare). Purified schizonts were incubated in CM at 37°C with fresh RBCs for 1-4 hours in a shaking incubator. Any remaining schizonts were removed by a second Percoll purification to leave tightly synchronised ring-stage parasites.

### Human Cells

Human RBCs were acquired from the National Health Service Blood and Transplant (NHSBT) service, Colindale, London, UK.

### Magnetic Purification

Components for the magnet rack were designed using Autodesk Inventor software and printed with an Objet30 3D printer using VeroGray resin. Strong 1-inch neodymium magnets (K&J magnetics) were inserted into the rack, which was then sealed. CS-MACS (Miltenyi Biotec) columns are filled with RPMI from a 2-way stopcock then inserted into the magnet-holder. Columns were washed with 10mL RPMI before use, then attached to 23 gauge blunt-ended syringe filters to control flow speed. The RBC suspension was added to the column at a hematocrit of under 20%, then washed with RPMI. Once the flowthrough ran clear, the columns were washed with an additional 20mL of RPMI. The columns were removed from the magnet and placed in 15mL tubes, and late-stage iRBCs were eluted with 10mL of RMPI, with the syringe tips still attached. Purity of both the elution and the flowthrough were checked by giemsa smear. If parasitemia in the elution was under 90%, the columns were returned to the magnet-holder and the eluate passed through one more time, with 10mL RPMI for washing, before a final elution with 10mL RPMI. Columns were washed with 100mL H2O followed by 10mL 70% EtOH, then dried and stored in a 37°C incubator.

### Mass spectrometry

Cell culture, lysis and protein digestion - across all experiments, iRBCs were enriched by magnet purification at 40-46hpi for *P. falciparum* iRBCs and ~24hpi for *P. knowlesi* iRBCs, to a purity of between 95-99% parasitemia. Flowthrough uRBC samples contained <1% infected cells. Samples were immediately lysed in ice cold 8M urea in 50mM HEPES pH8.5, supplemented with protease (Complete mini, Roche) and phosphatase (Phos Stop, Roche) inhibitors, and snap frozen in liquid nitrogen for storage at -80°C. Once thawed, samples were further solubilized by sonication (30% duty cycle, 3 x 30 seconds bursts, on ice). Protein concentration was then calculated using a BCA protein assay kit (Pierce), first diluting 50uL sample aliquots from all lysates 1:3 in 50mM ammonium bicarbonate to reduce the concentration of urea, and then following the instructions included in the kit. Lysates (1 mg each) were subsequently reduced with 5mM DTT for 30 minutes at 56°C and alkylated in the dark with 14mM iodoacetamide for 30 minutes at RT. Following iodoacetamide quenching with 5mM DTT for 15 minutes in the dark, lysates were diluted with 50mM ammonium bicarbonate to < 4M urea, and digested with LysC (Promega) for 2-3 hours at 37°C. Lysates were further diluted with 50mM ammonium bicarbonate to < 2M urea and digested with trypsin (Promega) at 1:50 (enzyme:protein) overnight at 37°C.

Sep-Pak desalting - samples were acidified with trifluoroacetic acid (TFA) (Thermo Fisher Scientific) to a final concentration of 0.4% (v/v) and left on ice for 10 minutes. All insoluble material was removed by centrifugation (4000 rpm, 10 minutes, 4°C) and supernatants were desalted on Sep-Pak lite C18 cartridges (Waters) in conjunction with a vacuum manifold. Columns were first washed with 3mL acetonitrile, conditioned with 1mL of 50% acetonitrile, 0.5% acetic acid in H2O, then equilibrated with 3mL of 0.1% TFA in H2O. The acidified samples were loaded, then desalted with 3mL of 0.1% TFA in H2O, washed with 1mL of 0.5% acetic acid in H2O then finally eluted with 1.2mL of 50% acetonitrile, 0.5% acetic acid in H2O. Each sample was then dried by vacuum centrifugation.

TMT labelling – Samples were dissolved at 1 mg/mL in 50mM Na-Hepes, pH 8.5 and 30% acetonitrile (v/v) and labelled with respective TMT reagents (Thermo Fisher Scientific, 2.5mg reagent/1mg sample) for 1 hour at RT. Labelling was then quenched with 0.3% hydroxylamine for 15 minutes at RT and samples acidified (pH~2) with formic acid. Subsequently, 2 μL aliquots of each labelled sample were mixed in 200 μL of 1% formic acid, stage tipped (see the Stage tip section below), and a test LC-MS/MS run was performed on a Q-Exactive mass spectrometer (see the LC-MS/MS section below) to verify labelling efficiency. If the reporter intensity ratios between samples were < 1.5, the lysates were mixed in a 1:1 ratio, vacuum dried and desalted on Sep-Pak C18 cartridges (2 columns/10mg protein) as described above.

Strong cation exchange (SCX) fractionation – for experiments 1, 2 and 3 SCX fractionation was performed prior to phosphopeptide enrichment. Peptides were resuspended in 400μL of 10mM ammonium formate pH 3, 25% acetonitrile by sonication and all insoluble material was removed by centrifugation. Samples were loaded on a 20 cm Polysulfoethyl-A column (4.6 mm inner diameter, 5 μm particle size, PolyLC) and fractionated using Agilent 1200 (Agilent) HPLC with a binary buffer system (solvent A: 10 mM ammonium formate pH 3, 25% acetonitrile; solvent B: 500mM ammonium formate pH 6.8, 25% acetonitrile) at a flow rate of 0.8mL/minute. The samples were run on a linear gradient of 0-60% B in 20 minutes and 60-100%B in 5 minutes with a total run time of 40 minutes including column conditioning. A total of 12 fractions were collected and vacuum dried.

Phosphopeptide enrichment – samples were solubilized in 1mL of loading buffer (80% acetonitrile, 5% TFA, 1M glycolic acid) and mixed with 5mg of TiO2 beads (Titansphere, 5 μm GL Sciences Japan). Samples were incubated for 10 minutes with agitation, followed by a 1 minute 2000 × g spin to pellet the beads. The supernatant was removed and used for a second round of enrichment as explained below. Beads were washed with 150μL of loading buffer followed by two additional washes, the first with 150μL 80% acetonitrile, 1% TFA and the second with 150μL 10% acetonitrile, 0.2% TFA. After each wash, beads were pelleted by centrifugation (1 minute at 2000 × g) and the supernatant discarded. Beads were dried in a vacuum centrifuge for 30 minutes followed by two elution steps at high pH. For the first elution step, beads were mixed with 100μL of 1% ammonium hydroxide (v/v) and for the second elution step with 100μL of 5% ammonium hydroxide (v/v). Each time beads were incubated for 10 minutes with agitation and pelleted at 2000 × g for 1 minute. The two elutions were removed following each spin, and subsequently pooled together before undergoing vacuum drying.

The supernatant from the TiO2 enrichment was desalted on two Sep-Pak columns and the High Select Fe-NTA phosphopeptide enrichment kit (Thermo Fisher Scientific) was used according to manufacturer’s instructions for a second round of enrichment. The supernatant containing all non-phosphorylated peptides (total proteome) was removed and stored at -80°C.

High pH sample fractionation – for experiments 4, 5 and 6 combined TiO2 and Fe-NTA phosphopeptide eluates were fractionated using the Pierce High pH Reversed-Phase kit (Thermo Fisher Scientific) according to manufacturer’s instructions.

Stage tip desalting – all samples were desalted prior to LC-MS/MS using Empore C18 discs (3M). Briefly, each stage tip was packed with one C18 disc, conditioned with 100μL of 100% methanol, followed by 200μL of 1% TFA. The sample was loaded in 100μL of 1% TFA, washed 3 times with 200μL of 1% TFA and eluted with 50μL of 50% acetonitrile, 5% TFA. The desalted peptides were vacuum dried in preparation for LC-MS/MS analysis.

LC-MS/MS and data processing – Samples were resuspended in 0.1% TFA and loaded on a 50 cm Easy Spray PepMap column (75 μm inner diameter, 2 μm particle size, Thermo Fisher Scientific) equipped with an integrated electrospray emitter. Reverse phase chromatography was performed using the RSLC nano U3000 (Thermo Fisher Scientific) with a binary buffer system (solvent A: 0.1% formic acid, 5% DMSO; solvent B: 80% acetonitrile, 0.1% formic acid, 5% DMSO) at a flow rate of 250 nL/minute. The samples were run on a linear gradient of 5-60% B in 150 minutes (Lumos) or 2-35% in 155 minutes (Q-Exactive) with a total run time of 180 minutes including column conditioning. The nanoLC was coupled to mass spectrometers using an EasySpray nano source (Thermo Fisher Scientific). The Orbitrap Lumos was operated in data-dependent mode using Xcalibur software. MS2 and MS3 acquisition methods were adapted from those described previously^77^. Briefly, for the MS2 method, HCD MS/MS scans (R=50,000) were acquired after an MS1 survey scan (R=120, 000) using MS1 target of 4E5 ions, and MS2 target of 2E5 ions. The number of precursor ions selected for fragmentation was determined by the “Top Speed” acquisition algorithm with a cycle time of 3 seconds, and a dynamic exclusion of 60 seconds. The maximum ion injection time utilized for MS2 scans was 86 ms and the HCD collision energy was set at 38. For the MS3 method, CID MS/MS scans (R=30,000) were acquired after an MS1 survey scan with parameters as above. The MS2 ion target was set at 5E4 with multistage activation of the neutral loss (H3PO4) enabled. The maximum ion injection time utilized for MS2 scans was 80 ms and the CID collision energy was set at 35. HCD MS3 scan (R=60,000) was performed with synchronous precursor selection enabled to include up to 5 MS2 fragment ions. The ion target was 1E5, maximum ion injection time was 105 ms and the HCD collision energy was set at 65. The Q-Exactive was operated in data-dependent mode acquiring HCD MS/MS scans (R=35,000) after an MS1 survey scan (R=70, 000) on the 10 most abundant ions using MS1 target of 1E6 ions, and MS2 target of 2E5 ions. The maximum ion injection time utilized for MS2 scans was 120 ms, the HCD normalized collision energy was set at 33 and the dynamic exclusion was set at 30 seconds. The peptide match and isotope exclusion functions were enabled.

Acquired raw data files were processed with MaxQuant ^78^ (version 1.5.2.8) and peptides were identified from the MS/MS spectra searched against *Plasmodium falciparum*, *Plasmodium knowlesi* (PlasmoDB, 2018 ^72^) and *Homo sapiens* (UniProt, 2018) proteomes using Andromeda ^79^ search engine. TMT based experiments in MaxQuant were performed using the ‘reporter ion MS2 or MS3’ built-in quantification algorithm with reporter mass tolerance set to 0.003 Da. Cysteine carbamidomethylation was selected as a fixed modification. Methionine oxidation, acetylation of protein N-terminus, deamidation (NQ) and phosphorylation (S, T, Y) were selected as variable modifications. The enzyme specificity was set to trypsin with a maximum of 2 or 3 missed cleavages depending on the experiment. The precursor mass tolerance was set to 20 ppm for the first search (used for mass re-calibration) and to 4.5 ppm for the main search. ‘Match between runs’ option was enabled (time window 0.7 min) for fractionated samples. The datasets were filtered on posterior error probability (PEP) to achieve a 1% false discovery rate on protein, peptide and site level.

### Data analysis

Mass spectrometry data sets were first input into Perseus ^80^ for annotation of protein names and the organism or origin. The data were filtered to remove common contaminants and IDs originating from reverse decoy sequences. To generate a list of all quantified phosphosites reporter intensities were filtered for 1 valid value. Subsequent data analysis was done using R programing in R studio. General-usage R packages used were readxl, xlsx, matrixStats, gridExtra, VennDiagram, ggplot2, matrixStats, gplots, RColorBrewer, and svglite.

The TMT label intensity values were log2 transformed for each experiment. The data were normalized by median ratio normalization, which is appropriate for count data ^81^. Briefly, the mean log2 intensities were calculated for each phosphopeptide where values were observed across all ten samples in a given experimental run (mean of each row). This row mean was then subtracted from each original log2 intensity in a given row. A median was taken of each column of this data to get a scaling factor for each sample, which was then subtracted from the original log2 intensities in a given column to get the normalized intensities. For experiment 1, phosphosites from Human, *P. falciparum*, and *P. knowlesi* proteins were normalized using a scaling factor calculated from Human RBC phosphosites only, as this was assumed to remain approximately constant for each sample. For experiments 2, 3, 4, 5, and 6, the scaling factor was calculated individually for Human and *P. falciparum* phosphosites, to account for any differences in the purity of iRBC between samples.

For experiment 1, the TMT label intensities for each sample were averaged across technical replicates and Log2 Fold Change values were calculated by pairwise comparisons of Human phosphosites between *P. falciparum* and *P. knowlesi* iRBCs, between *P. falciparum* iRBCs and uRBCs, and between *P. knowlesi* iRBCs and uRBCs. Log2 fold change values and -Log10 *P*-values (Welch's two-tailed t-tests assuming a gaussian distribution, n=3) were calculated individually for each of the three mass spectrometry runs, then averaged. Phosphosites were considered significantly changing if they exceeded a non-linear threshold based on the log2 fold change values and the *P*-values. The threshold was defined as:

$$y=\frac{c}{|x|-x_{0}}$$

Where:
y: -log10 *P*-value
x: log2Fold Change
x0: log2Fold change threshold (set to 1.5)
c: curvature constant (set to 1.5)

A discordance (DISCO) plot of *P. knowlesi* iRBCs and *P. falciparum* iRBCs was plotted with the log2 fold changes of PK iRBCs-uRBCs against PF iRBCs-uRBCs. DISCO scores for each phosphosite were calculated using ^82^:

DISCOscore=(log⁡2FCPF−log⁡2FCPK)×(−log⁡10PvaluePF+−log⁡10PvaluePK)

For experiment 2, we investigated the effect of different donors and blood type on phosphorylation within the RBC. A one-way ANOVA test was performed in R (function ‘aov’) on human phosphosites across both uninfected and infected samples. A gaussian distribution was assumed for the samples. Additional ANOVA tests were performed and correlation coefficients calculated to test for differences between blood types in the phosphorylation of uRBC proteins, the log2 fold change in RBC proteins between uRBCs and *P. falciparum* iRBCs, and the phosphorylation of *P. falciparum* proteins (Extended Data Figure 1). Correlation plots are also shown for each comparison (Extended Data Figure 1).

For experiments 3,4,5, and 6, Log2 fold change values were calculated between DMSO and RAP-treated samples within each experiment, then averaged between the replicates for FIKK1, FIKK4.1, FIKK4.2, FIKK10.2, and FIKK11 cKO lines. The significance threshold was set at 4* standard deviation of the Log2 fold change values for a given FIKK kinase. For experiment 4 and 5, the Log2 fold change between the NF54 parental line and the *P. knowlesi* iRBC line was also calculated for Human proteins only. Violin plots were created using the R package ‘ggplots2’. Proteins were annotated as exported if they have an exportpred score > 1 (PlasmoDB ^72^), or are annotated with Go-component terms which contained the words ‘host cell’ or ‘maurer’s cleft’. The ‘heatmap.2’ function from the R package ‘gplots’ was used to create heatmaps which included all sites which were observed across experiments 3, 4, 5, and 6, and which were phosphorylated less upon deletion of at least one FIKK kinase as these are likely to be true substrates. Sites (rows) are clustered by the complete linkage method with Euclidean distance measure.

**Extended Data Fig. 10:**
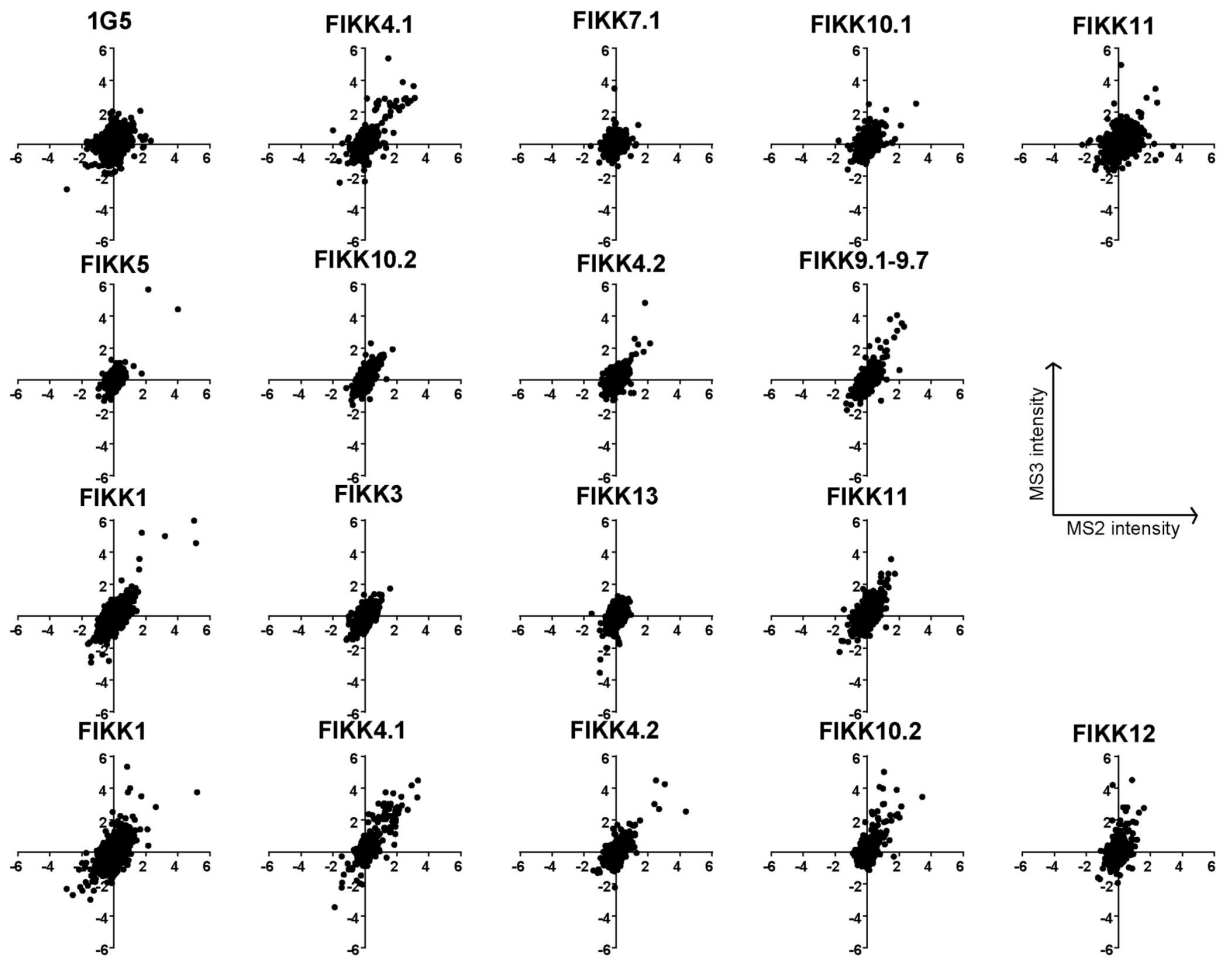
Correlation between the MS2 and MS3 mass-spectrometry methods. Intensity of peptides which are observed by both methods (approximately 50% of all detected peptides), with MS2 intensities on the x axis and MS3 intensities on the y axis.

A comparison between the MS2 and MS3 methods for experiments 3,4,5, and 6 showed that was a good agreement in Log2 fold change values between the different techniques (Extended Data Figure 10). While the MS2 method had greater coverage (approximately 2x higher than MS3), there was a higher background to noise ratio for MS3. Only approximately 50% of peptides were detected by both methods, indicating that the use of two different techniques greatly increased the coverage of the proteome.

### Unenriched Proteome Data Analysis for the Comparison of Parasite Growth Rates

The reporter intensity for each protein was calculated by perseus ^80^ from the average reporter intensity of all peptides on a protein. Proteins for which only 1 peptide was detected were removed as these were more variable between samples (MS/MS count). The data was filtered and Log2 transformed as described above for the phosphopeptide data, and normalised by median subtraction. The Log2 Fold change in intensity was calculated for each protein between the DMSO and RAP-treated lines, and this data was added to the phosphoproteome data in Supplemental table S4. To establish whether there are differences in growth between the lines, late-stage proteins were selected which were transcribed 3x more at 40hpi compared to 35hpi, based on RNA seq data from Toenhake *et. al*. on PlasmoDB ^1,72^(Table S2E). The Log2 fold change in the intensity of these proteins between each sample and the average of all other samples in the experiment (excluding *P. knowlesi* samples) was calculated. The same was done for all other proteins in the dataset (those not expressed specifically in late-stage schizonts) and this was subtracted from the Log2 fold change for late–stage proteins to control for general changes to protein abundance. Additionally, the log2 fold change between RAP and DMSO-treated FIKK cKO lines for late-stage proteins was also calculated in the same way.

### Plasmids and Parasite Transfection

Plasmids for the cKO lines were constructed as described previously ^32^ with some changes. Recodonised kinase domains with loxP introns were initially purchased from IDT as ‘custom genes’ inserted into a plasmid for FIKK4.1, FIKK7.1, FIKK8, FIKK10.1, and FIKK11. Subsequently, recodonised kinase domains fused to 3x HA but without the loxP introns were ordered as gblocks from IDT (Table S6). For cKO lines integrated using selection-linked integration (All FIKKs apart from FIKK9s, FIKK12 and FIKK10.1 in NF54), the 5’ homology arm, loxP intron, and the recodonised kinase domain with HA tag were PCR-amplified from *P. falciparum* NF54 genomic DNA, IDT ‘custom genes’, or IDT gblocks, respectively. Fragments were inserted by Gibson assembly into a previously-made pARL-based plasmid containing the T2A skip peptide, neomycin resistance cassette, second loxP intron, and GFP, which was digested at BglII and SalI restriction sites. The plasmid also contained a WR resistance cassette (for selection in the parasite) and an Ampicillin resistance cassette (for selection in *E. coli*). 100ug of the plasmid was obtained by midiprep from *E. coli* top 10 cells and transfected into highly synchronized 48hpi schizonts (either 1G5 or NF54) using an Amaxa electroporator and Lonza 4D-Nucleofector kit with P3 Primary cell buffer. Transfected lines were drug-selected after 48 hours, first with 2.5 nM WR99210 (Jacobus Pharmaceuticals) until iRBCs were visible by giemsa smear, then with 225μg/ml G418 (Gibco by LifeTechnology) to ensure correct integration into the FIKK locus.

For single FIKK cKO lines integrated by CrispR/Cas9, the rescue plasmid was constructed by PCR amplifying the 5’ homology region, loxP intron, recodonised kinase domain-3xHA, T2A-neomycin-loxPintron-GFP sequence, and 3’ homology region, then using Gibson assembly to insert all fragments into a pMK-RQ plasmid (IDT) containing a Kanamycin resistance cassette. 60 ug of this plasmid was digested by EcoRI, which was then heat-denatured at 65°C for 30 minutes. The digested plasmid was then combined with 20ug of a PDC2 plasmid containing Cas9 under a CAM promoter, the tracR RNA under a U6 promoter, and a hDHFRuFCU resistance cassette for positive selection with WR and negative selection by ancotil. The guide RNA sequence was inserted into bbsII cleavage sites in the tracR RNA sequence by Gibson assembly. Schizonts were transfected as described above and selected after 24h with 2.5 nM WR99210, which was added daily for 4 days. Once iRBCs were visible by giemsa smear, iRBCs were further selected with 225μg/ml G418.

For the FIKK9.1-FIKK9.7 deletion, a 9-fragment Gibson assembly was performed to create the rescue plasmid. This combined the FIKK9.1 5’ homology region, recodonised FIKK9.1-3xHA tag- loxP intron-T2A sequence (ordered from Geneart, see Table S6), Neomycin cassette, FIKK9.1 3’ homology region, FIKK9.7 5’ homology region, recodonised FIKK9.7-loxP sequence (ordered from Geneart), FIKK9.5 3’ homology arm, and a pMK-RQ plasmid (IDT), which was digested by HindIII and NcoI. 60ug of the plasmid was digested by EcoRI. The Crispr/Cas9 plasmid contained guides against both FIKK9.1 and FIKK9.7, which were first inserted individually into the cas9 plasmid as described above. The U6 promoter, tracR RNA and FIKK9.1 guide were then PCR amplified and inserted by Gibson assembly into the SalI site of the plasmid containing the FIKK9.7 guide RNA. 20ug of this plasmid was combined with the 60ug of digested rescue plasmid, and transfected into purified schizonts. Transfected parasites were selected after 24h with 2.5 nM WR99210 for 4 days.

Correct integration of all transfectants was confirmed by PCR (see Table S6 for primers). Parasite lines were cloned as described ^36^. The concentration of iRBCs was calculated and diluted into a suspension of complete media at 0.75% hematocrit, to approximately 500 parasites/mL. A 3-fold serial dilution series was dispensed into the wells, from 100 to 0.1 parasites per well in 200uL. After between 10-14 days, plaques per well were counted, and any wells containing a single plaque were transferred to round-bottomed 96-well plates and supplemented with fresh blood to 2% hematocrit. After approximately 14 days, the wells were checked by giemsa smear and samples from parasite-positive wells were then taken for PCR analysis to check for correct integration of the plasmids. 4 wells containing the desired insertion were transferred to T25 flasks, and one clone was then selected for all further experiments. For all experiments, ring-stage parasites were treated with either 100nM Rapamycin or DMSO for 4 hours, and excision was confirmed by PCR (Extended Data Figure 2 and Extended Data Figure 3).

### Plaque assay assessment of parasite growth

Parasite lines were tightly synchronized to a 4-hours window using Percoll (GE Healthcare). Synchronous parasite cultures at ring stage were treated with either DMSO or Rapamycin followed by 3 washes with Complete Medium (CM). Plaque assays were performed as previously described by Thomas *et al.* ^36^. DMSO- and RAP-treated parasites were plated in the same flat-bottomed 96-well plates (Corning 3596) using the central 60 wells of the plate, 30 for DMSO- and 30 for RAP-treated culture (10 parasites per well, 200μL CM, 0.75% Haematocrit). Evaporation was limited by adding sterile PBS to the outer wells. Plates were incubated in gassed chamber (C.B.S Scientific Culture Chamber M-312) filled with parasite gas (90% N2, 5% CO2, 5% O2) for 12 days. Plates were imaged using a Perfection V750 scanner (Epson) in top-down transmission light mode, saving images as 4,800dpi TIFF files. Wells of interest were selected using the Magic Wand tool (tolerance setting = 75) of Adobe Photoshop CC 2018. Finally, number of plaques and the plaque area for each well were quantified using the ‘Analyse Particles’ tool in Fiji (Size = 10-750 pixels; Circularity = 0.2-1.00). Graphpad Prism version 8 was used for statistical analysis of plaque assay data by unpaired welch’s *t*-test assuming a Gaussian distribution. The number of cells counted is shown in Extended Data Figure 4. A *P*-value of <0.05 was considered statistically significant.

### Flow Cytometry analysis of parasite growth

For parasitemia measurements by flow cytometry, parasites were tightly synchronised to a 2-hour window by Percoll synchronisation. Cultures were adjusted to approximately 0.5% parasitemia and 4% haematocrit in triplicate in 96 well plates and treated with either DMSO or Rapamycin for 4h. 20uL samples were taken every 24h and fixed in 2% paraformaldehyde/0.2% glutaraldehyde in PBS. Samples were stained with Sybrgreen or Hoechst 33342 and counted by flow cytometry on a LSRFortessa flow cytometer (Becton Dickinson) using FACSDiva software. An example of the gating strategy is shown in Extended Data Figure 9E. Data were analysed using FlowJo10 analysis software (Becton Dickinson). The experiment was repeated three times but only one replicate is shown. Statistical analysis was performed using t-tests with the Holm-Sidak method for multiple comparisons in Graphpad Prism.

### Immunoprecipitation and Western blot

FIKK7.1 HA-expressing parasites were lysed in 5ml RIPA buffer (ThermoFischer Scientific) by incubation on ice for 20-30 minutes. 100μl of anti-HA affinity matrix (Sigma-Aldrich) per condition was washed 3 times in RIPA buffer. 1ml of parasite lysate was spun at 13000rpm for 30 minutes at 4°C. Supernatants from lysates were mixed together with washed anti-HA affinity matrices and left to incubate at 4°C for 2-3 hours on a rotating wheel. Matrices were washed 3 times in RIPA buffer, boiled for 10 minutes in protein loading buffer and bound proteins were recovered in the supernatant after centrifugation.

All other western blot samples were obtained from Percoll-enriched schizonts resuspended in PBS, solubilised in protein loading buffer and denatured at 95°C for 10 minutes.

Parasite extracts and immunoprecipitation samples were subjected to SDS-PAGE, transferred to Transblot^®^ Turbo^™^ Mini-size nitrocellulose membranes (BIORAD) and blocked overnight in 5% skimmed milk in PBS/0.2%Tween-20 at 4°C. For integration and excision checks (Figure 2C), membranes were probed with rat anti-HA high affinity antibodies (Clone 3F10, Roche) (1:1000) and rabbit anti-MAHRP1 antibodies (1:2000) followed by incubation with relevant secondary fluorochrome-conjugated antibodies (Donkey anti-rabbit IRDye 680LT (LI-COR) (1:20000); Goat anti-rat IRDye 800CW (LI-COR) (1:20000)). For western blots investigating adducin S726 phosphorylation (Figure 3D), membranes were probed with rat anti-HA high affinity antibodies (Roche (1:1000)), rabbit anti-MAHRP1 (a gift from Julian Rayner and Lindsay Parish) (1:2000), B-12 mouse anti-spectrin (SantaCruz Biotechnology (1:10000)), rabbit anti-dematin (Invitrogen (1:1000)), mouse anti-αadducin (Abcam (1:2000)) and rabbit anti-αadducin p726 (Abcam (1:1500)). The same secondary fluorochrome-conjugated antibodies were used as above in addition to goat anti-mouse IRDye 680LT or 800CW (LI-COR) (1:20000).

Antibody reactions were carried out in 5% skimmed milk in PBS/0.2%Tween-20 and washed in PBS/0.2%Tween-20. Antigen-antibody reactions were visualized using the Odyssey Infrared Imaging system (LI-COR Biosciences, Nebraska, United States).

### Immunofluorescence assay

22x22mm coverslips (VWR International) were coated with 200μl of Concanavalin A (Santa Cruz Biotechnology, 5mg/ml in water) for 20 minutes in a humid chamber at 37°C. In the meantime, iRBCs from culture were rinsed twice with 1ml of pre-warmed PBS by centrifugation in at 2000rpm for 2 minutes and diluted to 1% haematocrit still in pre-warmed PBS. Coverslips were washed with 200μl of pre-warmed PBS and 200μl of the 1% haematocrit solution was added onto the coverslips. After 15 minutes incubation at 37°C, unbound cells were gently washed away with PBS and remaining cells were fixed with freshly made 2% paraformaldehyde in PBS for 20 minutes at room temperature. For the IFAs in Extended Data Figure 5, thin smears of parasite culture were frozen at -80 °C before thawing and washing with PBS and fixing with 4% PFA and 0.0075% glutaraldehyde. For all IFAs, the fixative was removed and fixed cells were rinsed 3 times with PBS. Cells were permeabilised with 0.1% Triton^®^ X-100 diluted in PBS for 20 minutes followed by 3 washes with PBS. Cells were blocked with 3% BSA in PBS for 1 hour at room temperature. Labelling of the cells was performed for at least 1 hour at room temperature with primary antibody diluted in PBS 1% BSA: high affinity anti-HA (Clone 3F10, Roche) (1:1000); anti-MAHRP1 (1:1000) (gift from Julian Rayner and Lindsay Parish); or anti-KAHRP (1:1000) (EMRR, donated by Dr. Jana McBride). After 3 washes with PBS, coverslips were incubated with relevant Alexa Fluor secondary antibodies (1:2000 in PBS 1% BSA) at room temperature for 1 hour, in the dark. After 3 final washes with PBS, coverslips were mounted in Prolong^®^ Gold antifade reagent (Invitrogen) containing the DNA dye 4’, 6-diamidino-2-phenylindole (DAPI) and sealed with nail polish. Images were taken using a Ti-E Nikon microscope using a 100x TIRF objective at room temperature equipped with a LED-illumination and an Orca-Flash4 camera. Images were processed with Nikon Elements software (Nikon, Japan).

### Network

An undirected network was constructed in Cytoscape ^83^ with the 13 FIKK kinases as the main nodes connected to other nodes that represent RBC and exported *P. falciparum* phosphoproteins. Betweenness centrality of each node was calculated and the network was shown in a force-directed layout with some manual arrangement, with nodes with degree of 1, i.e. proteins that only connect to one FIKK kinase, removed. The thickness of the connecting line represents the number of sites significantly changing on each protein due to each kinase, while lines are coloured according to the average log2 fold change of all the significantly-changing sites. Symbol size represents the number of connections (degree) to each protein.

### Motif-x

All phosphorylation sites identified to be dependent for a given FIKK were analyzed for a specific phosphorylation motif using rmotif-x ^84^ using the standard parameters. All phosphorylation sites identified in all experiments were set as background and phosphorylation sites that were reduced in phosphorylation state upon FIKK deletion were used as the foreground dataset.

### Recombinant FIKK4.1 expression and purification

The DNA sequence coding for *Plasmodium falciparum* 3D7 FIKK4.1 (PlasmoDB ID: PF3D7_0424500 ^72^) kinase domain residues Y215-N622 was codon optimised for *E. coli* expression (IDT ^®^) and inserted into pET-28a vector (Novagen^®^) to produce a N-terminal Thrombin cleavable His_6_-tag fusion (MGSS**HHHHHH**SSGLVPRGSH*MASMTGGQQMG*RGS, where the sequence in bold is the His_6_-tag, underlined sequence is the Thrombin site and the sequence in italics is the T7-tag). The insert sequence was verified by DNA sequencing. For expression in *E.coli*, BL21-Gold (DE3) cells (Stratagene,^®^) were transformed with pET-28-FIKK4.1 and grown over day at 30°C in ZYM-505 media supplemented with 50μg/ml Kanamycin. Prior to adding IPTG, the temperature in the incubator was dropped to 20°C and protein expression was induced by adding 0.5mM IPTG. Cultures were grown at 20°C for a minimum of 16h after which cells were harvested by centrifugation. In a typical preparation, 10g of cells were resuspended in 100ml lysis buffer (50mM Tris-HCl pH7.5, 500mM NaCl, 1mM TCEP, 20mM Imidazole, 10mM MgS0_4_, 10% Glycerol, 2 protease inhibitor cocktail tablets (cOmplete, EDTA free, Roche^®^), lysed by sonication and clarified by centrifugation at 20,000 x g for 30 minutes at 4°C. The supernatant was loaded into an 1ml HisTrap column (GE HealthcareC^®^) and bound proteins were eluted in 50mM Tris-HCl pH7.5, 500mM NaCl, 1mM TCEP, 300mM Imidazole, 10% Glycerol. After concentration, the sample was loaded on a Hi-Load Superdex 200 16/600 column (GE Healthcare^®^) equilibrated in 50mM Tris-HCl pH7.5, 250mM NaCl, 1mM TCEP, 10% Glycerol. The fractions containing FIKK4.1 were analysed by SDS-PAGE.

### *In Vitro* Kinase activity assay

Recombinant FIKK4.1 kinase activity was measured using the ADP-Glo™ Kinase Assay (Promega ^®^) quantifying the amount of ADP produced during the kinase reaction. Briefly, the kinase reaction was conducted at room temperature for 1 hour by mixing 100nM of recombinant FIKK4.1 with 10μM of ATP and with or without 10μM of substrate (Po, PIESP2__267, PTP4_1091, KAHRP_345, or PKC PEP) in 40μl of kinase buffer (100mM MOPS, 50mM magnesium chloride and 50mM manganese chloride, pH 7.4). 40μl of ADP-Glo™ Reagent was added to stop the kinase reaction and deplete the unconsumed ATP. After incubation at room temperature for another hour, 80μl of kinase detection reagent was added and incubated for 30 minutes at room temperature. Luminescence was measured using the multi-mode microplate reader FLUOstarn^®^ Omega. The results were statistically tested with an ordinary one-way ANOVA test plus a Tukey’s multiple comparison test comparing the mean of each condition with the mean of every other condition. The data presented are as means±SEM, n=3 biological replicates including 3 technical replicates each.

### Membrane contour detection and flickering spectrometry

*Plasmodium falciparum* NF54 and FIKK 4.1 parasite cultures treated with DMSO and treated with RAP were synchronised to a one hour window by Percoll (GE Healthcare) before imaging. Then parasites were diluted in culture medium (RPMI-1640 supplemented with HEPES, 40 mM; D-glucose, 10 mM; glutamine, 2 mM; gentamicin sulphate, 25 mg/L; AlbumaxII, 0.5 % vol/vol) (Sigma) at 0.01% haematocrit and transferred in SecureSeal Hybridization Chambers (Sigma-Aldrich). A custom-built temperature control system was used to maintain the optimal culture temperature of 37°C during imaging experiments. The sample was placed in contact with a transparent glass heater driven by a PID temperature controller in a feedback loop with the thermocouple attached to the glass slide. A Nikon Eclipse Ti-E inverted microscope (Tokyo, Japan) was used with a Nikon 60X Plan Apo VC, N.A. 1.40, oil immersion objective, kept at physiological temperature through a heated collar. Motorized functions of the microscope were controlled via custom software written in-house. Videos were acquired for around 20 seconds in bright field using a CMOS camera (model GS3-U3-23S6M-C, Point Grey Research/FLIR Integrated Imaging Solutions (Machine Vision), Ri Inc., Canada) at 514 frames/s and 0.8 ms exposure time. For each condition (NF54 + DMSO, NF54 + RAP, FIKK 4.1 + DMSO, FIKK 4.1 + RAP), parasites were recorded at 20, 24, 28, 32, and 36 hours post invasion by two experimentalists using two microscopes at the same time. Recordings of uninfected erythrocytes were also taken in the same period of time. Data from two biological replicates were analysed, and the number of cells counted for each condition are summarised in Table S7. Significant variances were calculated with unpaired *t*-tests assuming Gaussian distribution in Graphpad Prism version 8.

To obtain biophysical parameters such as bending modulus, tension, radius, and viscosity of cells, we employed the flickering spectroscopy technique. First, erythrocyte contours were detected for each frame with subpixel resolution by an optimised algorithm developed in house and implemented in Matlab (The MathWorks, Natick, MA). Contour detection of infected erythrocytes after 36 hours was difficult due to the uneven, non-uniform equatorial shape of the cells caused by the development of the parasite inside. Detailed explanations of the contour detection algorithm and the membrane fluctuation analysis are extensively described in previous works ^48,85,86^. Briefly, the contour was decomposed into fluctuation modes by Fourier transform to give a fluctuation power spectrum of mean square mode amplitudes at the cell equator 〈|*h*(*q_x_,y* = 0)|^2^〉 as a function of mode wave vector *q_x_*. The bending modulus and tension *σ* can then be fitted using the following equation:

(1) $$\langle {\left|h\left(q_{x},y=0\right)\right|}^{2}\rangle =\frac{1}{L}\frac{k_{B}T}{2\sigma }\left(\frac{1}{q_{x}}-\frac{1}{\sqrt{\frac{\sigma }{k}+q_{x}^{2}}}\right),$$

where *k_B_* is the Boltzmann constant, *T* is temperature, and *L* is mean circumference of the erythrocyte contour. This model considers only cell fluctuations at the equatorial plane. From Eq. 1 we extracted bending modulus, tension, and equatorial radius of cells. When fitting the fluctuation data, we considered modes 5-14 in which Eq. 1 was a good representation of the spectrum. Low mode numbers (< 5) were excluded due to significant influence of the cell shape and high mode numbers (>14) had noise in the spectra. In this range, Eq. 1 has limiting behaviours: tension dominates at low mode, 〈*h*(*q_x_, y* = 0)^2^〉~*q* ^−1^ for $\left(\sigma \gg \kappa q_{x}^{2}\right);$ instead, bending modulus dominates at high q, 〈*h*(*q_x_, y* = 0)^2^〉~*q* ^−3^ for $\left(\sigma \ll \kappa q_{x}^{2}\right).$ Examples of flickering spectra are reported in Extended Data Figure B.

The dynamics of the fluctuations can also be quantified ^48^. We calculated the autocorrelation function of the modes, averaged over 10000 frames. This analysis worked well on modes 7 to 11, higher modes decayed too fast to be fitted. Since we only image the contour of the cell, and therefore probe only modes as a function of their mode component *q_x_*, the timescales of decay of this function are called and can be fitted by:

(2) $$C_{q_{x}}(t)={\langle h_{q_{x}}\left(t^{\prime }\right)h_{q_{x}}\left(t^{\prime }+t\right)\rangle }_{t^{\prime }}=\frac{\int dq_{y}\langle h\frac{2}{q}\rangle e^{\left(-t/\tau_{\vec{q}}\right)}}{\int dq_{y}\langle h\frac{2}{q}\rangle },$$

where the timescale *τ_q_x__* is in general given by

(3) $$\frac{1}{\tau_{qx}}=\frac{2\gamma +\sigma q^{2}+\kappa q^{4}}{2\left(\frac{\eta M}{R^{2}}+q\eta_{int}+q\eta_{ext}\right)}.$$

Eq. 3 gives the relaxation timescale of the modes of a two-dimensional membrane, as a function of the wave vectors; *η_M_* is the two-dimensional membrane viscosity, *η_int_* and *η_ext_* are the viscosities of the fluid on either side of the membrane, *R* is the radius of the cell membrane. Two-dimensional phospholipid bilayers have viscosities *η_M_* < 10^−9^ N s m^−1^ for temperatures above room *T*, and *η_ext_* ≌ 10^−3^ Pa s, therefore *η_int_* dominates the denominator of Eq.3, i.e. we are able to measure from this procedure the internal viscosity of the RBC. Finally, from *τ;_qx_*, using the values of κ and σ obtained from the static spectrum of the same cell in Eq. 1, we then obtained the value of the internal viscosity of the RBC.

### Microsphiltration

Parasites were synchronized to a 1h window then treated with Rapamycin/DMSO at ring stages. At 30-35 hpi in the next cycle when parasitemia was approximately 5%, the two samples were divided into two 1mL samples and diluted to 2% haematocrit, then stained with either Hoechst 33342 (NEB) diluted 1:2000 or SybrGreen diluted 1:10000, for 30 minutes at 37°C. After washing 3x with 1mL PBS, RAP and DMSO-treated lines were mixed 1:1 in duplicate – one Hoechst-RAP:Sybrgreen-DMSO, and one Sybrgreen-RAP:Hoechst-DMSO. The microsphiltration tips were prepared as described previously ^50^. 150mg of calibrated metal microspheres (96.50% tin, 3.00% silver, and 0.50% copper; Industrie des Poudres Sphériques) of the 2 different size distributions (5–15 μm or 15–25 μm in diameter) were combined together and resuspended in 1mL RPMI. This was quickly resuspended then pipetted onto the filter of an inverted pipette tip (VWR), which had approximately 1.5cm from the tip cut off. This was connected to a 2-way stopcock by inserting tubing fittings into the tip to create an air-tight seal, and connecting with plastic tubing. The stopcock was connected to a 20mL syringe containing RPMI, which was placed in an electric pump (*kd*Scientific, model Legato 110). To wash the beads, 5mL of RPMI was perfused through the tip at 1mL/min. With a 1mL syringe, 600uL of parasite culture was inserted into the tip using the 2-way stopcock. 6mL of RPMI was then perfused through the tip at a flow rate of 1ml/min. Both the upstream (before microsphiltration) and downstream (flowthrough) were fixed in 4% paraformaldehyde/0.2% glutaraldehyde in PBS for 1h. Samples were immediately analysed by flow cytometry to prevent mixing of the dyes, using a BD LSR Fortessa cell analyser. Hoechst fluorescence was detected using a 355nm (UV) excitation laser with a 450/50nm bandpass filter, while Sybrgreen fluorescence was detected with a 488nm (blue) excitation laser, a 505nm longpass filter and a 530/30nm bandpass filter. An example of the gating strategy is shown in Extended Data Figure 9D. The number of infected cells stained by each dye was analysed by FlowJo, and a before/after ratio calculated for both the RAP and DMSO parasites in each of the duplicates. Due to variation between tips, retention by the beads in the RAP/DMSO- treated NF54 and FIKK4.1 lines was compared in paired ANOVA multiple comparison analyses for each tip individually with the Greenhouse-Geissier correction for non-sphericity and Sidak correction for multiple comparisons, n=10 including two technical replicates across five biological replicates.

### qRT-PCR

RNA from NF54 parental wild type line and transgenic FIKK4.1 synchronized ring stage parasites was extracted with Trizol following the manufacturer’s instructions (RNeasy Minikit, Qiagen). cDNA synthesis was performed by random primers after DNase I treatment (TURBO DNase, Ambion) using the Super Script III First Stand Synthetis system (from Invitrogen). Primers pairs to analyze *var* gene expression have been described previously ^87^. Quantitative real time PCR reactions were performed on a CFX 96 thermocycler (Biorad). Transcriptional level of each *var* gene was normalized using the house keeping control gene *seryl tRNA transferase* (PlasmoDB ID: PF3D7_0717700 ^72^).

### Cytoadhesion assay

Circles large enough to accommodate a sample size of 20μl were drawn at the bottom of a Petri dish using a delimiting Dako pen (Agilent Technologies). 20μl of CSA (Sigma-Aldrich) (1mg/ml in PBS) or 1% BSA control (BSA Fraction V, Sigma-Aldrich) in PBS were added to each spot and left to incubate for at least 3 hours at 4°C. Spots were then washed 3 times with PBS and blocked with 1% BSA in PBS for an hour at room temperature. After 3 additional washes with PBS, 20μl of a suspension of magnet purified trophozoite iRBCs (30-34hpi) at 1.10^6^ parasites/ml in PBS was applied to each spot and allowed to settle for one hour at room temperature. Unbound RBCs were washed off the Petri dish by gently adding 25ml of PBS to the centre of the Petri dish followed by 2 minutes rocking on an orbital shaker (50rpm). After 5 washes, bound cells were fixed with 2% glutaraldehyde in PBS for at least 2 hours at 4°C. Glutaraldehyde was removed from the Petri dish with a final wash with PBS. Phase contrast images of spots were collected using an EVOS™ XL inverted microscope. Bound cells were counted using the Analysis Particles tool in Fiji (size = 10 – 200 pixels, circularity = 0.2 – 1.00). The results were statistically tested with a two-way ANOVA test plus a multiple comparison Sidak test comparing all means in GraphPad Prism^®^ 8. The data presented are as mean±SEM, n = 5 biological replicates, each with at least two technical replicates, which were averaged before statistical analysis.

### SEM

Parasites were synchronized to a 1h window then RAP/DMSO-treated at ring stage. 5 days after treatment at ~40hpi, iRBCs were purified by magnetic enrichment. Approximately 10 x 10^6 iRBCs were fixed in 2.5% gluteraldehyde / 4% formaldehyde in PBS for 1h at 37°C, then washed 2x with PBS and resuspended in 200uL PBS. 13mm coverslips were coated with Poly-L lysine for 15 minutes then washed with dH2O and left to dry for 1h. 50uL of the iRBCs suspension was added to the dry coverslips and allowed to settle for 10 minutes. Unattached cells were removed by gentle washing with 2x PBS then 2x dH2O. iRBCs were dehydrated by washing with 70% EtOH, 90% EtOH, then 100% EtOH for 5 minutes each, without allowing the cells to dry. The coverslips were submerged in Acetone then dried in a Leica EM CPD300 critical point drier. Coverslips were attached to stubs then coated with 4nm of platinum using a Quorum 150R S sputter coater, then imaged using a Phenom ProX scanning tunneling microscope.

### Electron micrographs of knobs

3 ul of mature FIKK4.1 KO schizonts (50 % haematocrit) were applied to a glow-discharged (35 mA/30 sec) carbon-coated, copper (200 mesh) finder grid (EMS) and incubated at room temperature for 1 min. Grids were blotted to remove excess cells and washed twice in Tris-buffered saline (TBS) (Sigma), blotting after each wash. Cells were lysed by passing the grid in and out of the meniscus of a drop of 30x diluted TBS for 1 min. After blotting excess buffer, grids were stained with 2 % (w/v) uranyl acetate before blotting dry. Wild-type 3D7 cells were prepared using the same method, but 10 um gold fiducials were also applied to the grids prior to staining. Grids were mounted on a Model 2040 dual-axis tomography holder (Fischione Instruments) and membrane patches were located at low magnification using a Tecnai T12 120 kV electron microscope (FEI) equipped with a 4kx4k Ultrascan 4000 CCD camera (Gatan). Dual-axis tilt series were acquired at a magnification of x42000 (2.56 Å/pixel) from -60° to +60° with an increment of 2° using SerialEM ^88^. Processing was carried out using the eTomo workflow from IMOD ^89–91^. Images were aligned using either patch tracking or tracking of the gold fiducials for the FIKK4.1 KO or wild-type 3D7 samples, respectively. Tomograms were reconstructed by back-projection with 15 rounds of simultaneous iterative reconstruction technique-like (SIRT-like) filtering. Non-anisotropic diffusion filtering (K=50, iterations=25) was applied to the final trimmed tomograms. Averages of 5 Z slices were produced using Slicer in 3dmod ^90^.

### VAR2CSA surface expression

Cytoadhesion experiments and VAR2CSA surface expression by flow cytometry were performed on RAP/DMSO treated and magnet purified parasites. Cells were diluted to 3x 10.6 cells/ml and 100uL of each sample was distributed into 96-well plates, including one unlabeled and secondary-only control. iRBCs were washed with PBS then resuspended in 1% BSA in PBS for 1h. The plate was centrifuged at 1200rpm for 1 minute, the supernatant was removed and iRBCs were resuspended in 50uL of Rabbit anti-VAR2CSA antibody diluted 1/100 in 1% BSA in PBS, and agitated at 50rpm for 1h. After centrifugation and 2x washes with 1% BSA in PBS, the iRBCs were resuspended in 50uL of anti-rabbit PE diluted 1/100 in 1% BSA in PBS and kept in the dark for 1h. iRBCs were washed twice with PBS before fixing with 4% paraformaldehyde, 0.2% glutaraldehyde in PBS for 1h. After washing, parasites were stained with Hoechst (1:1000 in PBS) for 30 minutes before flow cytometry using high-throughput acquisition on a BD LSR Fortessa cell analyser. PE fluorescence was detected with a 561nm (yellow) excitation laser and a 586/15 bandpass filter. Using FlowJo software, the population was first gated on single cells based on the side and forward scatter, then on Hoechst-positive infected parasites, before the median fluorescent intensity of the PE-fluorescence was calculated for each line. An example of the gating strategy for infected cells is shown in Extended Data Figure 9E. Due to the variation in fluorescence intensity between different experiments, the ratio of RAP/DMSO fluorescence intensity was calculated for each experiment (n=4 for FIKK4.1 and FIKK10.2, n=3 for NF54), and the ratio for NF54, FIKK4.1 and FIKK10.2 compared by Sidak-corrected multiple comparison ANOVA using Graphpad Prism version 8.

## Extended Data

## Supplementary Material

Supplementary information

## Figures and Tables

**Table 1:** Proteins identified as FIKK4.1 substrates previously shown to play a role in malaria pathogenesis. The gene ID is indicated along with the protein name, the role previously reported in the literature and the positions of the significantly changing phosphosites with the L2FC in parentheses.

FIKK4.1 parasite Substrate	Gene name	Role previously reported in the literature	Site identified (Log2 FC)
PF3D7_0731100	PTP2	Cell-Cell communication (Regev-Rudzki *et al.*, 2013) PfEMP1 surface presentation (Maier et al., 2008)	231(3.03); 255(2.59); 257(2.54); 159(2.04); 498(1.59); 279(1.29)
PF3D7_1478600	PTP3	PfEMP1 surface presentation and iRBC rigidification (Maier *et al.*, 2008)	673(1.85)
PF3D7_0730900	PTP4	PfEMP1 surface presentation (Maier *et al.*, 2008)	1198(2.70); 1091(2.66); 2098(2.41); 1677(2.38); 2082(2.24); 1906(2.02); 2097(1.91); 1197(1.88); 1972(1.86); 1485(1.83); 1884(1.52); 1486(1.49); 334(1.27); 1495(1.26)
PF3D7_1401300	EH2	Cytoadherence and iRBC rigidification (da Silva *et al.*, 2016) Vasoactivity (Spillman *et al.*, 2016)	111(2.48)
PF3D7_0831700	Hsp-70x	PfEMP1 surface presentation, Cytoadherence and iRBC rigidification (Charnaud *et al.*, 2016)	110(2.90); 296(2.23); 108(2.09); 114(1.22)
PF3D7_0501200	PFE60 (PIESP2)	Maurer's cleft architecture and Pf332 localisation (Zhang *et al.*, 2018)	267(3.35)
PF3D7_0202000	KAHRP	Knob formation and Cytoadherence (Crabb *et al.*, 1997) PfEMP1 surface presentation (Horrocks *et al.*, 2005) iRBC rigidification (Glenister *et al.*, 2002)	345(2.48); 348(2.18); 344(1.46); 347(1.20); 634(1.05)
PF3D7_0424600	Plasmodium exported protein (PHISTb)	Cytoadherence and Knob formation (Maier *et al.*, 2008)	124(1.75)
PF3D7_0201900	*Pf*EMP3	PfEMP1 surface presentation (Waterkeyn *et al.*, 2000) iRBC rigidification (Glenister *et al.*, 2002)	72(1.36)
PF3D7_0632400	Rifin	Cytoadherence and Rosetting (Goel *et al.*, 2015)	69(1.31)
**FIKK4.1 human Substrate**	**Gene name**	**Role previously reported in the literature**	**Site identified (Log2 FC)**
Q9UEY8	Gamma-adducin	Components of the RBC cytoskeleton (Salomao *et al.*, 2008)	651(2.64)
Q08495	Dematin		85(2.51); 81(1.51); 87(1.29)
Q00013	MPP1/p55		344(2.28)
P11171	Protein 4.1		84(1.99); 551(1.76)
P23276	Kell		25(1.59); 15(1.34)
P35611	Alpha-adducin		482(1.30)

## Data Availability

The mass spectrometry proteomics data have been deposited to the ProteomeXchange Consortium via the PRIDE^92^ partner repository with the dataset identifier PXD015833. Gene sequences and annotations for *P. falciparum* 3D7 and *P. knowlesi* strain H were acquired from PlasmoDB.org (2018) ^72^, and human sequences were acquired from Uniprot.org (2018). RNA sequencing data from Toenhake *et. al.* ^1^ was also used. Source data in the form of unprocessed gels and western blots corresponding to figures 2c and 3d, and extended data figures 2 and 3 are available with the article.

## Code availability

No custom code deemed central to the conclusions to this manuscript has been used in this study.
