# Supplementary material for: An Exported Kinase Family Mediates Species-Specific Erythrocyte Remodelling and Virulence in Human Malaria
Source: Nat Microbiol. Author manuscript; Available in PMC 2020 Oct 23. (PMC7116245; doi:10.1038/s41564-020-0702-4)
Supplement: Supplementary information [file EMS85986-supplement-Supplementary_information.pdf]

Supplementary Information – Additional Discussion

Phosphorylation of RBC proteins upon invasion by *P. falciparum* has been documented previously using a combination of immunoblotting and mass spectrometry techniques[1-3]. We provide a quantitative comparison between uRBC and iRBC cultured under the same conditions. Our analysis verified some previously observed changes in phosphorylation, but also identified many additional changes to phosphosites which may influence RBC characteristics (see supplementary table T3E). As expected, using MS2 and MS3 mass spectrometry methods on two different instruments increased the coverage of the parasite phosphoproteome, with 54.4% of confidently assigned sites on *P. falciparum* proteins previously undocumented (PlasmoDB). We observed good correlation between phosphorylation sites identified by both methods, increasing the confidence in our data (supplemental figure 6).

Very few RBC sites were phosphorylated upon infection with *P. knowlesi*, however phosphosites were detected on several parasite proteins predicted to be exported. These may be substrates of host kinases or unknown exported kinases and may be involved in *P. knowlesi*-specific RBC adaptions such as the presentation of the antigenically variant SICAvars or the formation of cavaeolae. While *P. knowlesi*-infected RBC can also adhere to host receptors to cause severe disease[4], this process is not dependent on FIKKs and likely evolved after the divergence of the *Laverania*. Most sites which were specifically phosphorylated upon infection with *P. falciparum* were subsequently shown to be either direct or indirect substrates of the FIKK kinases, indicating that this family is the main driver of host cell phosphorylation for this species. While no other kinase in *P. falciparum* contains a PEXEL motif that would predict export, *P. falciparum* casein kinase I has been shown to be exported into the host cell, despite its lack of a signal peptide or transmembrane domain[5,6]. As this kinase is conserved in *P. knowlesi*, it could play a species-independent role.

Disruption of the kinase-substrate relationship is the most likely explanation for the reduction in phosphorylation observed upon deletion of most FIKKs. However, depletion or mislocalisation of proteins, as well as small differences in growth between RAP and DMSO-treated parasites may also play a part. For most substrates, however, the unenriched proteome data or the presence of other unchanging phosphosites on the same protein suggests such examples are likely a minority. For example, phosphorylation of residues on the megadalton protein Pf332 is affected on some, but not all phosphorylation sites. The increase in phosphorylation on several proteins upon deletion of some FIKKs

indicates that some indirect effects do occur, which is not unexpected. These FIKKs may regulate other kinases or phosphatases or FIKK deletion may result in protein mis-localisation allowing phosphorylation by other kinases. Using recombinant FIKK4.1, we showed that it is able to phosphorylate peptides corresponding to three of its predicted substrates, suggesting that in this case these are direct targets of the kinase.

While some FIKKs are predicted to be important for parasite propagation [7], a striking growth defect was observed only upon deletion of FIKK8, the non-exported kinase, which exhibited a 43% reduction in parasitemia compared to wild type after 120h. The function of FIKK8 and the underlying cause of the growth defect are not known, and we have not further pursued it here as FIKK8 is not exported. While small differences were observed in our growth curves for the other lines, for example FIKK10.1, this was not consistent between biological replicates and may be due to experimental factors such as differences in starting parasitemia. By using the stage specific expression of a subset of proteins between parasite lines as a molecular clock, we conclude that differences in growth between conditions for each parasite line unlikely explain any differences in phosphorylation state or phenotype. However, a small delay in growth may occur over longer time scales or under conditions not tested here.

While it is not currently possible to test the survival of genetically modified parasites in humans, the export of most FIKKs into the RBC suggests they are likely important for adapting to conditions within the host. The divergence of the Laverania clade is believed to have occurred approximately one million years ago [8], yet most FIKKs remain highly conserved between species, predicting distinct and important functions for each kinase[9]. Guided by our phosphoproteomic data, we chose to investigate the detailed mechanism of FIKK4.1 for this study, demonstrating its role in modulating both erythrocyte rigidity and PfEMP1-mediated cytoadhesion. The data also provides insights into the function of other FIKKs. For example, phosphorylation of PfEMP1 trafficking proteins was dependent on a number of other FIKKs in addition to FIKK4.1, suggesting that several FIKKs may act together to support this pathway. Indeed, deletion of FIKK4.2 was previously shown to reduce cytoadhesion through a defect in knob morphology [10]. Here we show that FIKK4.2 controls phosphorylation of KAHRP and the lysine-rich membrane-associated PHISTb protein (LYMP) which binds PfEMP1, providing a possible molecular mechanism for this phenotype [11,12]. While deletion of FIKK4.1 also alters the phosphorylation of KAHRP, we observed no striking differences in knob formation. As both FIKK4.1 and FIKK4.2 appear to be involved

in different aspects of PfEMP1 trafficking and presentation, deletion of several kinases simultaneously may result in a complete block in adhesion.

In addition to FIKK4.1, other FIKKs are also predicted to modulate erythrocyte rigidity. Previous deletion of FIKK4.2, FIKK7.1, and FIKK12 resulted in significantly more deformable RBCs. However, these FIKKs were not deleted conditionally and our rigidity experiments raise concerns regarding the variation in rigidity between individual cloned lines. By both flickering and microsphiltration analysis, the FIKK4.1 line was substantially more rigid than the parental NF54 line despite tight synchronisation. This may be explained by small differences in growth rates between parasite lines, which is supported by our molecular clock of stage specific proteins (Extended Data 6). In addition, differential expression of other rigidity-modulating genes such as STEVORs could play a role in the observed differences in rigidity between parasite lines[13]. The variation in retention rates between the replicate microsphiltration experiments further demonstrates that even a small difference in time post infection can have a large effect on RBC rigidity. This emphasises the strength of our conditional approach where the deletion of a FIKK kinase domain is tested in an otherwise isogenic parasite line; as all experiments were conducted within 72h of splitting samples for either RAP or DMSO treatments, differences between these lines are very likely to be due to FIKK4.1 deletion rather than lack of synchronisation between samples. The effect of FIKK4.1 on cell rigidity is likely due to phosphorylation of cytoskeletal components. For example, phosphorylation of dematin and protein 4.1, two of its predicted targets, is known to affect cytoskeletal rigidity [14,15]. Phosphorylation of dematin by recombinant FIKK4.1 *in vitro* was previously documented at position 110[16]. Here we observed no changes at this position and instead observed increased phosphorylation at positions 81, 85, and 87. This difference may be due to changes in the accessibility of the protein in *in vitro* assays with a recombinant kinase which lacks the N-terminal targeting region.

Phosphosites affected by deletion of FIKK10.2 and FIKK9s were predominantly located on proteins known to localise to Maurer's clefts. Deletion of several of these proteins, such as Pf332, REX1, MAHRP2, VCAP1, and SBP1 was previously shown to affect Maurer's cleft morphology[17], stacking[18,19], tethering to the cytoskeleton[20], and ability to traffic PfEMP1 to the RBC surface[21-23]. Interestingly, de-phosphorylation of the N-terminus of SBP1 was shown to be important for merozoite egress[24]. The FIKKs may therefore be involved in some of these processes. We episomally expressed the Maurer's cleft protein REX2 fused to mCherry to investigate the size and movement of the clefts in FIKK10.2 and

FIKK9s KO lines. None of the experiments yielded a clear phenotype, indicating that the function of these kinases is either more subtle, occurs at a specific time point, or lies elsewhere.

Some of the FIKK-dependent RBC phosphosites we identified were previously shown to be important for parasite invasion [25,26]. The interaction of *P. falciparum* proteins with their RBC ligands can trigger phosphorylation of the RBC cytoskeleton. Binding of EBA175 to Glycophorin A, or RH5 to Basigin induces phosphorylation of several sites on Glycophorin A and an increase in RBC deformability[26,270]. We observe changes in phosphorylation of several of these sites upon deletion of FIKK11 (either increasing or decreasing in intensity). FIKK11 may therefore control phosphorylation of these proteins after parasites have entered the cell, perhaps to prevent subsequent invasion or signalling events. Similarly, deletion of FIKK5 resulted in the loss of phosphorylation on beta spectrin at positions 1301 and 1444, the former of which is significantly phosphorylated upon contact with *P. falciparum* merozoites. As phosphorylation occurs even when invasion is blocked with heparin or the R1 peptide, host cell kinases were hypothesised to be responsible. However, as FIKK5 is localised in merozoites it may be secreted into the host cell upstream of the invasion-blocking effect of heparin and R1 to mediate phosphorylation. Alternatively, FIKK5 may be secreted into the RBC post-invasion to sustain phosphorylation of these residues throughout infection. This would be the first observation of a *Plasmodium* kinase that is secreted into the host cell during or shortly after invasion, as observed for rhoptry kinases in the related *Toxoplasma* parasites [28]. While most of the invasion-related sites on spectrin or Glycophorin A are not phosphorylated by *P. knowlesi*, position 130 on Glycophorin A is phosphorylated in cells infected by both species, possibly indicating a common requirement for invasion.

Some phosphorylation sites on the RBC cytoskeleton are well-studied. We show that the increase in phosphorylation of adducin at position 726 is a consequence of FIKK1 activity. Phosphorylation of this site is predicted to affect the actin-capping activity of adducin [29,30], and actin is known to be remodelled by the parasite [31,32]. It is unclear whether adducin is a direct substrate of FIKK1 or if the kinase activates host PKC, which is known to phosphorylate this site[29,30]. Other host cell kinases may also be activated or deactivated by FIKKs to modulate RBC phosphorylation; indeed casein kinase II [33], PKA[14,34], and PAK-MEK[35] are all implicated in modifying red blood cell properties.

The unique fingerprints of the FIKKs supports the hypothesis that each plays a defined role. The specificity of the kinases is likely due to their localisation within the cell, substrate specificities and stage-specific expression. Differences in localisation between the FIKKs is likely mediated by their divergent N-termini. The localisation of FIKK4.1 and FIKK4.2 at the RBC periphery is likely due to their basic repeats, as observed for similar sequences in other exported proteins [36]. FIKK7.1 also contains basic repeats predicted to interact with the periphery but we were unable to observe it by IFA due to low expression. Specific antibodies against the lowly expressed kinases may facilitate their localisation in the future. FIKK4.1 and FIKK1 are also palmitoylated [37], which may allow them to interact with the RBC membrane. Unlike their N-termini, the kinase domains of the FIKKs are relatively well-conserved. Despite this, only FIKK4.1 showed a strong preference for the R/KxxS/T phosphorylation motif. FIKK8 was shown to also phosphorylate a peptide containing a -3 arginine *in vitro* [38], however our recombinant FIKK4.1 was not able to phosphorylate the same peptide, indicating that subtle differences between the FIKKs may dictate their specificity for different substrates, allowing diversification of functions within this family of otherwise highly conserved kinases. Our analysis revealed no clear motif for the other FIKKs, potentially due to the lower number of differentially phosphorylated sites observed. Phosphosites containing an arginine at the -3 position are typical of PKA and PKC substrates and many proteins have therefore been predicted to be phosphorylated by these kinases [39], however the FIKKs may mimic these kinases in some cases. Recombinant kinases will help elucidate the distinct substrate preferences of the FIKKs in the future.

Several signalling events in the infected RBC may be time-dependent. All samples were collected at the late schizont stage as this is when the majority of erythrocyte phosphorylation occurs and our western blot analysis indicates that most FIKKs are present at this time [1,40,41]. We reasoned that this maximized the likelihood of identifying FIKK-specific targets, but as FIKKs display different expression patterns, several phosphorylation events may be missed [42]. Some FIKKs may also be important in other life stages; RBC rigidity and morphology are both important for the sequestration of *P. falciparum* gametocytes in the bone marrow during maturation, which is also unique to *Laverania* parasites [34,43,44], and liver-stage parasites may also benefit from interference of host-cellular signalling pathways by parasite exported kinases.

In summary we have shown that exported FIKK kinases are key players in the modification of the host cell. However, many questions remain on the mechanistic details of FIKK function, including their

activation mechanism, interactions with other kinases, and the consequences of phosphorylation on the substrates. The advent of RBC genetics may help elucidate the effect of RBC protein phosphorylation on the properties of the cell[45-47]. Further investigation of the role of FIKKs in other life stages and in field isolates may help explain their expansion in the *Laverania* and their role in severe malaria.

References

1 Pantaleo, A. *et al.* Analysis of changes in tyrosine and serine phosphorylation of red cell membrane proteins induced by P. falciparum growth. *PROTEOMICS* **10**, 3469-3479, doi:10.1002/pmic.201000269 (2010).

2 Wu, Y. *et al.* Identification of phosphorylated proteins in erythrocytes infected by the human malaria parasite Plasmodium falciparum. *Malaria Journal* **8**, 105, doi:10.1186/1475-2875-8-105 (2009).

3 Bouyer, G. *et al.* Plasmodium falciparum infection induces dynamic changes in the erythrocyte phospho-proteome. *Blood Cells, Molecules, and Diseases* **58**, 35-44, doi:10.1016/j.bcmd.2016.02.001 (2016).

4 Fatih, F. A. *et al.* Cytoadherence and virulence - the case of Plasmodium knowlesi malaria. *Malaria Journal* **11**, 33, doi:10.1186/1475-2875-11-33 (2012).

5 Batty, M. B., Schittenhelm, R. B., Doerig, C. & Garcia-Bustos, J. Interaction of Plasmodium falciparum Casein kinase 1 (PfCK1) with components of host cell protein trafficking machinery. *bioRxiv*, 617571, doi:10.1101/617571 (2019).

6 Dorin-Semblat, D. *et al.* Malaria Parasite-Infected Erythrocytes Secrete PfCK1, the Plasmodium Homologue of the Pleiotropic Protein Kinase Casein Kinase 1. *PLOS ONE* **10**, e0139591, doi:10.1371/journal.pone.0139591 (2015).

7 Zhang, M. *et al.* Uncovering the essential genes of the human malaria parasite Plasmodium falciparum by saturation mutagenesis. *Science* **360**, doi:10.1126/science.aap7847 (2018).

8 Otto, T. D. *et al.* Genomes of all known members of a Plasmodium subgenus reveal paths to virulent human malaria. *Nat Microbiol* **3**, 687-697, doi:10.1038/s41564-018-0162-2 (2018).

9 Sundararaman, S. A. *et al.* Genomes of cryptic chimpanzee Plasmodium species reveal key evolutionary events leading to human malaria. *Nature communications* **7**, 11078, doi:10.1038/ncomms11078 (2016).

10 Kats, L. M. *et al.* An exported kinase (FIKK4.2) that mediates virulence-associated changes in Plasmodium falciparum-infected red blood cells. *Int J Parasitol* **44**, 319-328, doi:10.1016/j.ijpara.2014.01.003 (2014).

11 Proellocks, N. I. *et al.* A lysine-rich membrane-associated PHISTb protein involved in alteration of the cytoadhesive properties of Plasmodium falciparum-infected red blood cells. *FASEB J* **28**, 3103-3113, doi:10.1096/fj.14-250399 (2014).

12 Oberli, A. *et al.* Plasmodium falciparum Plasmodium helical interspersed subtelomeric proteins contribute to cytoadherence and anchor P. falciparum erythrocyte membrane protein 1 to the host cell cytoskeleton. *Cellular Microbiology* **18**, 1415-1428, doi:10.1111/cmi.12583 (2016).

13 Sanyal, S. *et al.* Plasmodium falciparum STEVOR proteins impact erythrocyte mechanical properties. *Blood* **119**, e1-8, doi:10.1182/blood-2011-08-370734 (2012).

14 Koshino, I., Mohandas, N. & Takakuwa, Y. Identification of a Novel Role for Dematin in Regulating Red Cell Membrane Function by Modulating Spectrin-Actin Interaction. *Journal of Biological Chemistry* **287**, 35244-35250, doi:10.1074/jbc.M111.305441 (2012).

15 Manno, S., Takakuwa, Y. & Mohandas, N. Modulation of Erythrocyte Membrane Mechanical Function by Protein 4.1 Phosphorylation. *Journal of Biological Chemistry* **280**, 7581-7587, doi:10.1074/jbc.M410650200 (2005).

16 Brandt, G. S. & Bailey, S. Dematin, a human erythrocyte cytoskeletal protein, is a substrate for a recombinant FIKK kinase from Plasmodium falciparum. *Mol Biochem Parasitol* **191**, 20-23, doi:10.1016/j.molbiopara.2013.08.003 (2013).
17 Glenister, F. K. *et al.* Functional alteration of red blood cells by a megadalton protein of Plasmodium falciparum. *Blood* **113**, 919-928, doi:10.1182/blood-2008-05-157735 (2009).
18 Hanssen, E. *et al.* Targeted mutagenesis of the ring-exported protein-1 of Plasmodium falciparum disrupts the architecture of Maurer's cleft organelles. *Mol Microbiol* **69**, 938-953, doi:10.1111/j.1365-2958.2008.06329.x (2008).
19 McHugh, E. *et al.* A repeat sequence domain of the ring-exported protein-1 of Plasmodium falciparum controls export machinery architecture and virulence protein trafficking. *Mol Microbiol* **98**, 1101-1114, doi:10.1111/mmi.13201 (2015).
20 Pachlatko, E. *et al.* MAHRP2, an exported protein of Plasmodium falciparum, is an essential component of Maurer's cleft tethers. *Mol Microbiol* **77**, 1136-1152, doi:10.1111/j.1365-2958.2010.07278.x (2010).
21 Cooke, B. M. *et al.* A Maurer's cleft-associated protein is essential for expression of the major malaria virulence antigen on the surface of infected red blood cells. *The Journal of cell biology* **172**, 899-908, doi:10.1083/jcb.200509122 (2006).
22 Maier, A. G. *et al.* Skeleton-binding protein 1 functions at the parasitophorous vacuole membrane to traffic PfEMP1 to the Plasmodium falciparum-infected erythrocyte surface. *Blood* **109**, 1289-1297, doi:10.1182/blood-2006-08-043364 (2007).
23 McHugh, E. *et al.* The Plasmodium falciparum protein VCAP1 controls Maurer's cleft morphology, knob architecture and PfEMP1 trafficking. *bioRxiv*, 741033, doi:10.1101/741033 (2019).
24 Blisnick, T., Vincensini, L., Fall, G. & Braun-Breton, C. Protein phosphatase 1, a Plasmodium falciparum essential enzyme, is exported to the host cell and implicated in the release of infectious merozoites. *Cell Microbiol* **8**, 591-601, doi:10.1111/j.1462-5822.2005.00650.x (2006).
25 Zuccala, E. S. *et al.* Quantitative phospho-proteomics reveals the Plasmodium merozoite triggers pre-invasion host kinase modification of the red cell cytoskeleton. *Sci Rep* **6**, 19766, doi:10.1038/srep19766 (2016).
26 Sisquella, X. *et al.* Plasmodium falciparum ligand binding to erythrocytes induce alterations in deformability essential for invasion. *Elife* **6**, doi:10.7554/eLife.21083 (2017).
27 Aniweh, Y. *et al.* P. falciparum RH5-Basigin interaction induces changes in the cytoskeleton of the host RBC. *Cellular Microbiology* **19**, e12747, doi:10.1111/cmi.12747 (2017).
28 Boothroyd, J. C. & Dubremetz, J.-F. Kiss and spit: the dual roles of Toxoplasma rhoptries. *Nat. Rev. Microbiol.* **6**, 79-88, doi:10.1038/nrmicro1800 (2008).
29 Matsuoka, Y., Hughes, C. A. & Bennett, V. Adducin Regulation DEFINITION OF THE CALMODULIN-BINDING DOMAIN AND SITES OF PHOSPHORYLATION BY PROTEIN KINASES A AND C. *Journal of Biological Chemistry* **271**, 25157-25166, doi:10.1074/jbc.271.41.25157 (1996).
30 Matsuoka, Y., Li, X. & Bennett, V. Adducin Is an In Vivo Substrate for Protein Kinase C: Phosphorylation in the MARCKS-related Domain Inhibits Activity in Promoting Spectrin–Actin Complexes and Occurs in Many Cells, Including Dendritic Spines of Neurons. *The Journal of Cell Biology* **142**, 485-497, doi:10.1083/jcb.142.2.485 (1998).
31 Cyrklaff, M. *et al.* Hemoglobins S and C interfere with actin remodeling in Plasmodium falciparum-infected erythrocytes. *Science (New York, N.Y.)* **334**, 1283-1286, doi:10.1126/science.1213775 (2011).
32 Rug, M. *et al.* Export of virulence proteins by malaria-infected erythrocytes involves remodeling of host actin cytoskeleton.

33 Hora, R., Bridges, D. J., Craig, A. & Sharma, A. Erythrocytic casein kinase II regulates cytoadherence of Plasmodium falciparum-infected red blood cells. *J. Biol. Chem.* **284**, 6260-6269, doi:10.1074/jbc.M809756200 (2009).
34 Naissant, B. *et al.* Plasmodium falciparum STEVOR phosphorylation regulates host erythrocyte deformability enabling malaria parasite transmission. *Blood* **127**, e42-53, doi:10.1182/blood-2016-01-690776 (2016).
35 Sicard, A. *et al.* Activation of a PAK-MEK signalling pathway in malaria parasite-infected erythrocytes. *Cellular Microbiology* **13**, 836-845, doi:10.1111/j.1462-5822.2011.01582.x (2011).
36 Davies, H. M., Thalassinos, K. & Osborne, A. R. Expansion of Lysine-rich Repeats in Plasmodium Proteins Generates Novel Localization Sequences That Target the Periphery of the Host Erythrocyte. *J. Biol. Chem.* **291**, 26188-26207, doi:10.1074/jbc.M116.761213 (2016).
37 Jones, M. L., Collins, M. O., Goulding, D., Choudhary, J. S. & Rayner, J. C. Analysis of protein palmitoylation reveals a pervasive role in Plasmodium development and pathogenesis. *Cell Host & Microbe* **12**, 246-258, doi:10.1016/j.chom.2012.06.005 (2012).
38 Osman, K. T. *et al.* Biochemical characterization of FIKK8--A unique protein kinase from the malaria parasite Plasmodium falciparum and other apicomplexans. *Mol Biochem Parasitol* **201**, 85-89, doi:10.1016/j.molbiopara.2015.06.002 (2015).
39 Lasonder, E. *et al.* The Plasmodium falciparum Schizont Phosphoproteome Reveals Extensive Phosphatidylinositol and cAMP-Protein Kinase A Signaling. *Journal of Proteome Research* **11**, 5323-5337, doi:10.1021/pr300557m (2012).
40 Murray, M. C. & Perkins, M. E. Phosphorylation of erythrocyte membrane and cytoskeleton proteins in cells infected with Plasmodium falciparum. *Molecular and Biochemical Parasitology* **34**, 229-236, doi:10.1016/0166-6851(89)90051-0 (1989).
41 Pease, B. N. *et al.* Global Analysis of Protein Expression and Phosphorylation of Three Stages of Plasmodium falciparum Intraerythrocytic Development. *Journal of Proteome Research* **12**, 4028-4045, doi:10.1021/pr400394g (2013).
42 Foth, B. J. *et al.* Quantitative time-course profiling of parasite and host cell proteins in the human malaria parasite Plasmodium falciparum. *Mol. Cell Proteomics* **10**, M110.006411, doi:10.1074/mcp.M110.006411 (2011).
43 Tiburcio, M. *et al.* A switch in infected erythrocyte deformability at the maturation and blood circulation of Plasmodium falciparum transmission stages. *Blood* **119**, e172-180, doi:10.1182/blood-2012-03-414557 (2012).
44 Aingaran, M. *et al.* Host cell deformability is linked to transmission in the human malaria parasite Plasmodium falciparum. *Cell Microbiol* **14**, 983-993, doi:10.1111/j.1462-5822.2012.01786.x (2012).
45 Satchwell, T. J. *et al.* Genetic manipulation of cell line derived reticulocytes enables dissection of host malaria invasion requirements. *Nature communications* **10**, 1-9, doi:10.1038/s41467-019-11790-w (2019).
46 Dankwa, S. *et al.* Genetic Evidence for Erythrocyte Receptor Glycophorin B Expression Levels Defining a Dominant Plasmodium falciparum Invasion Pathway into Human Erythrocytes. *Infection and Immunity* **85**, e00074-00017, doi:10.1128/IAI.00074-17 (2017).
47 Kanjee, U. *et al.* CRISPR/Cas9 knockouts reveal genetic interaction between strain-transcendent erythrocyte determinants of Plasmodium falciparum invasion. *PNAS* **114**, E9356-E9365, doi:10.1073/pnas.1711310114 (2017).

NF54::DiCre	uRBC	20hpi	24hpi	28hpi	32hpi	36hpi
tension	39	48/50	49/49	46/46	45/48	46/46
bending modulus	39	34/37	33/38	28/26	20/22	23/17
viscosity	39	48/50	49/49	46/46	45/48	46/46
radius	39	48/50	49/49	46/46	45/48	46/46
**FIKK4.1::DiCre**						
tension	102	87/95	90/89	87/95	90/98	94/86
bending modulus	102	65/71	71/53	47/52	42/51	41/50
viscosity	126	76/100	94/84	88/100	104/102	100/96
radius	102	87/96	90/89	88/95	91/98	94/87

Supplementary Information Table 1: Sample size for flickering analysis (DMSO/RAP) - The number of cells counted for each condition, over each time point, for all the lines analysed by flickering analysis over two biological replicates.

	P- value					
**NF54 lines**	**0 hpi**	**24 hpi**	**48 hpi**	**72 hpi**	**96 hpi**	**120 hpi**
FIKK1 DMSO vs. FIKK1 RAP	>0.9999	>0.9999	>0.9999	>0.9999	>0.9999	>0.9999
FIKK3 DMSO vs. FIKK3 RAP	>0.9999	>0.9999	>0.9999	>0.9999	>0.9999	>0.9999
FIKK4.1 DMSO vs. FIKK4.1 RAP	>0.9999	>0.9999	>0.9999	>0.9999	>0.9999	>0.9999
FIKK4.2 DMSO vs. FIKK4.2 RAP	>0.9999	>0.9999	>0.9999	>0.9999	>0.9999	0.0831
FIKK5 DMSO vs. FIKK5 RAP	>0.9999	>0.9999	>0.9999	0.9995	>0.9999	0.3781
FIKK7 DMSO vs. FIKK7 RAP	>0.9999	>0.9999	>0.9999	>0.9999	>0.9999	>0.9999
FIKK8 DMSO vs. FIKK8 RAP	>0.9999	>0.9999	0.0782	<0.0001	<0.0001	<0.0001
FIKK9.1-9.7 DMSO vs. FIKK9.1-9.7 RAP	>0.9999	>0.9999	>0.9999	>0.9999	0.956	0.833
FIKK10.1 DMSO vs. FIKK10.1 RAP	>0.9999	>0.9999	>0.9999	>0.9999	0.3781	0.3781
FIKK10.2 DMSO vs. FIKK10.2 RAP	>0.9999	>0.9999	>0.9999	>0.9999	>0.9999	>0.9999
FIKK11 DMSO vs. FIKK11 RAP	>0.9999	>0.9999	>0.9999	>0.9999	0.9943	0.5354
FIKK12 DMSO vs. FIKK12 RAP	>0.9999	>0.9999	>0.9999	>0.9999	>0.9999	0.9977
FIKK13 DMSO vs. FIKK13 RAP	>0.9999	>0.9999	>0.9999	>0.9999	<0.0001	>0.9999
NF54 DMSO vs. NF54 RAP	>0.9999	>0.9999	>0.9999	>0.9999	>0.9999	>0.9999
**1G5 lines**						
FIKK4.1 DMSO vs. FIKK4.1 RAP	>0.9999	>0.9999	NA	0.7733	0.8972	0.9938
FIKK7.1 DMSO vs. FIKK7.1 RAP	>0.9999	0.9988	NA	>0.9999	0.8972	>0.9999
FIKK10.1 DMSO vs. FIKK10.1 RAP	>0.9999	>0.9999	NA	>0.9999	0.3564	0.0153
FIKK11 DMSO vs. FIKK11 RAP	>0.9999	0.9994	NA	0.9964	0.9671	0.9845

Supplementary Information Table 2: p- values for growth curves of FIKK knockout lines (see extended data 4). p - values were calculated by ANOVA using the Tukey method to correct for multiple comparisons. N=3 biological replicates.

NF54::DiCre DMSO vs RAP	20hpi	24hpi	28hpi	32hpi	36hpi
bending modulus	0.3962	0.8350	0.0349	0.8147	0.8856
viscosity	0.0361	0.1895	0.5054	0.9514	0.2865
radius	0.4814	0.9598	0.9842	0.8925	0.3567

FIKK4.1::DiCre DMSO vs RAP	20hpi	24hpi	28hpi	32hpi	36hpi
bending modulus	0.7934	0.2771	0.3016	0.7998	0.0552
viscosity	0.1951	0.1209	0.9808	0.8897	0.2216
radius	0.1900	0.7429	0.1196	0.3648	0.3065

Supplementary Information Table 3: p- values for flickering analysis (see extended data 8) - P-value over each time point, for all the lines analysed by flickering analysis. Statistical significance was determined by a two-tailed *t*-test. N= 2 biological replicates. The p-values for tension are in Figure 5 legend**.**
